# Intracellular pH regulates β-catenin with low pHi increasing adhesion and signaling functions

**DOI:** 10.1016/j.jbc.2026.111134

**Published:** 2026-01-07

**Authors:** Brandon J. Czowski, Angelina N. Marchi, Katharine A. White

**Affiliations:** 1 Department of Chemistry and Biochemistry, University of Notre Dame, Notre Dame, Indiana, USA; 2 Harper Cancer Research Institute, University of Notre Dame, South Bend, Indiana, USA

**Keywords:** beta-catenin, Wnt signaling, adherens junctions, protein degradation, confocal microscopy, intracellular pH, pH regulation, plakoglobin

## Abstract

Intracellular pH (pHi) dynamics are linked to cell proliferation, migration, and differentiation. The adherens junction (AJ) and signaling protein β-catenin has decreased abundance at high pHi due to increased proteasomal-mediated degradation. However, the effects of low pHi on β-catenin abundance and function have not been characterized. Here, we use population-level and single-cell assays to show that low pHi stabilizes β-catenin, increasing junctional, cytoplasmic, and nuclear abundance. We assayed single-cell protein degradation rates to show that β-catenin half-life is longer at low pHi and shorter at high pHi compared to control. Importantly, a constitutively stabilized and pHi-insensitive β-catenin mutant (β-catenin-H36 R) has a longer and pHi-independent half-life. We also determined that the pH-dependent stability of β-catenin affects both its adhesion and signaling functions. We show that the composition of AJs changes with pHi; at low pHi, E-cadherin-containing AJs are enriched in β-catenin while plakoglobin abundance is reduced. Conversely, when β-catenin is lost from E-cadherin-containing AJs at high pHi, plakoglobin is increased. We also found that cell area was reduced at low pHi and increased at high pHi compared to control while cell volume was unaffected, suggesting pHi alters cell–cell adhesion. Finally, we show that low pHi increases β-catenin transcriptional activity in single cells and is indistinguishable from a Wnt-on state, while high pHi reduces β-catenin transcriptional activity compared to control cells. This work characterizes pHi as a true rheostat regulating β-catenin abundance, stability, and function, solidifying β-catenin as a molecular mediator of pHi-dependent cell processes *via* pH-dependent adhesion and signaling functions.

Spatiotemporal intracellular pH (pHi) dynamics regulate cell processes, such as differentiation (1), cell migration (2), cell cycle progression (3), and cell fate determination (4). While low pHi is required to maintain the adult stem cell niche (1, 4) and transient increases in pHi are required for successful differentiation (1), the molecular drivers of these pHi-dependent processes are unknown. Recent work identified β-catenin as a pH-sensitive protein with decreased abundance at high pHi driven by pH-dependent binding of β-catenin to the E3 ligase, β-transducin repeat-containing protein (β-TrCP) (5).

β-catenin is a multifunctional protein with structural and transcriptional roles, maintaining adherens junctions (AJs) between epithelial cells and functioning as the main signal transducer of the Wnt pathway (6, 7, 8). Colocalization of β-catenin with E-cadherin at AJs preserves junction integrity (9), protects E-cadherin from degradation (10), and recruits α-catenin to link AJs to the cytoskeleton (11). In the absence of Wnt ligand, cytoplasmic β-catenin is phosphorylated by casein kinase 1 α and glycogen synthase kinase 3-β, enabling binding and ubiquitination by β-TrCP (12) for subsequent proteasomal degradation (13). Wnt activation inhibits the destruction complex (14): kinase activity is reduced, and β-TrCP is dissociated (15, 16). Wnt activation results in nonphosphorylated (active) β-catenin accumulating in the cytoplasm, translocating to the nucleus, and activating transcription of proliferative and morphogenic genes (17, 18, 19) by displacing Groucho in the repressive Groucho/TCF/LEF complex (20). The dual roles of β-catenin in AJs and Wnt signaling are essential for cell differentiation, stem cell maintenance, and tissue homeostasis (21, 22). Thus, the 2018 paper showing that high pHi decreases β-catenin stability (5) led several groups to subsequently suggest pH-dependent stabilization of β-catenin may underlie pH-dependent cell differentiation and tissue morphogenesis (4, 23).

However, while White *et al*. previously showed that high pHi reduces whole-cell and junctional β-catenin abundance in the *Drosophila* eye epithelia and in mammalian epithelial cells (5), the work had several significant limitations. First, the effect of decreased pHi on β-catenin abundance was not examined due to the lack of reliable methods for lowering pHi in epithelial cells. Second, the impact of pHi-mediated β-catenin abundance changes on AJ integrity and composition was not fully characterized. Third, the pH-dependent transcriptional activity of endogenous β-catenin was not investigated. Given the established context-dependent function of β-catenin, our understanding of how changes in pHi regulate β-catenin abundance and function remains incomplete. Thus, a full characterization of the effects of both high and low pHi on the adhesion and signaling roles of β-catenin is needed.

Here, we determined pH-dependent effects on β-catenin abundance, subcellular localization, degradation dynamics, and function using methods to both raise and lower pHi in Madin–Darby Canine Kidney cells. Using population-level immunoblots, we demonstrate pH-dependent β-catenin abundance with increased abundance at low pHi compared to high pHi. We also used immunofluorescent microscopy to characterize the effects of pHi on the abundance of distinct subcellular pools of β-catenin within single cells. We show that nuclear, cytoplasmic, and junctional β-catenin pools are all decreased at high pHi while low pHi stabilizes both the nuclear and cytoplasmic pools of β-catenin. We also determined β-catenin degradation dynamics in single cells using a photoconvertible mMaple3-β-catenin fusion. We show that low pHi increases β-catenin half-life while high pHi reduces its half-life. Furthermore, a constitutively stabilized β-catenin mutant (β-catenin H36 R), where the pH-sensitive His36 is mutated to a nontitratable arginine, exhibits a high and pH-insensitive half-life. Collectively, our data demonstrate that pHi functions as a rheostat to regulate β-catenin degradation rates.

Importantly, our work also characterizes how pHi regulates β-catenin function *via* altering AJ composition and transcriptional activation. At low pHi, β-catenin is enriched in E-cadherin-containing AJs, while high pHi depletes β-catenin from E-cadherin-containing AJs. However, recruitment of plakoglobin at high pHi maintains AJ integrity and epithelial morphology. We also found pH-dependent changes in cell shape but not volume, a phenotype normally associated with altered cell adhesion. We found that low pHi reduces cell cross-sectional area and high pHi increases cell cross-sectional area compared to control. Additionally, we found that modest (∼10%) increases in nuclear abundance of total β-catenin and active (nonphosphorylated) β-catenin at low pHi significantly increases β-catenin transcriptional activity to levels indistinguishable from those observed with addition of extracellular Wnt ligand. Our findings establish that pHi functions as a rheostat to regulate the stability and function of endogenous wildtype β-catenin. This work improves our understanding of the functional and biochemical outcomes of pH-dependent β-catenin regulation that may underlie both normal (differentiation) and pathological (transformation, cancer stem cells) processes marked by temporal changes in pHi (1, 24, 25).

## Results

### PHi differentially affects subcellular pools of β-catenin

To investigate the pH-dependent regulation of β-catenin in a mammalian cell model, we used Madin–Darby canine kidney (MDCK) cells. β-catenin has been extensively studied in MDCK cells, where it was previously shown that increased pHi was sufficient to reduce overall β-catenin abundance at the population level as well as nuclear and junctional pools at the single-cell level (5). This prior work established a role for high pHi in regulating β-catenin abundance; however, the lack of methods to lower pHi in this cell line prevented complete characterization of the role of pHi in regulating β-catenin abundance, localization, and function in epithelial cells.

**Figure 1:**
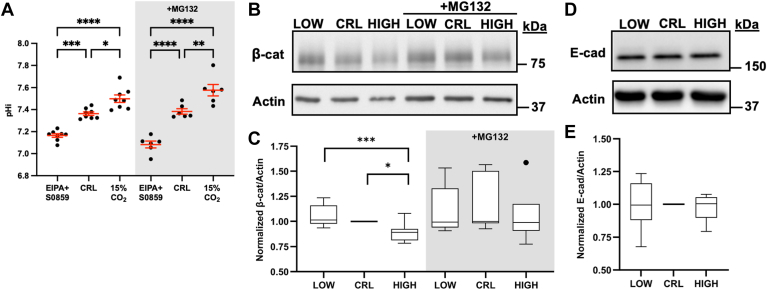
**Decreased pHi stabilizes β-catenin abundance through a proteosome-dependent mechanism**. *A*, Box and whisker plots showing population level pHi measurements in MDCK cells after 24-h treatments with 1 μM EIPA+ 30 μM S0859 (low) or 15% CO_2_ (high) to lower and raise pHi, respectively. Mean and SEM shown. n = 6 to 8 biological replicates. *B*, representative immunoblot of β-catenin (β-cat) and actin under low, control, and high pHi conditions in the presence and absence of proteosome inhibition (10 μM MG132). *C*, quantification of β-catenin immunoblot data collected as described in B. Individual biological replicates were normalized to control MDCK within each experiment. Box and whisker plots show median (*line*), 25th-75th percentile (*boxes*), min and max (*whiskers*), and outlier values with points. n = 6 to 8 biological replicates. *D*, representative immunoblot of E-cadherin and actin under low, control, and high pHi conditions. *E*, quantification of E-cadherin immunoblot data collected as described in *D* and displayed and normalized as described in *C*. n = 6 biological replicates. Statistical analyses in *A* were determined using one-way ANOVA test with Sidak’s correction for multiple comparisons. Statistical analyses in *C* and *E* were determined with ratio paired *t*-tests between treatment groups and one-sample *t*-tests with a hypothetical mean of 1.0 when comparing to control (which had no variation due to normalization). ∗*p* < 0.05; ∗∗*p* < 0.01 ∗∗∗*p* < 0.001; ∗∗∗∗*p* < 0.0001. EIPA, 5-(N-ethyl-N-isopropyl) amiloride; MDCK, Madin–Darby canine kidney; pHi, intracellular pH.

To experimentally lower pHi in MDCK cells, we used the combination of a sodium-proton exchanger 1 inhibitor (5-(N-ethyl-N-isopropyl)amiloride (EIPA)) and a sodium-bicarbonate cotransporter (NCBn1) inhibitor (2-chloro-N-[[2′-[(cyanoamino)sulfonyl][1,1′-biphenyl]-4-yl]methyl]-N-[(4-methylphenyl)methyl]-benzamide (S0859)) (26). To experimentally raise pHi, we used 15% CO_2_ atmospheric culture conditions, which increases the bicarbonate concentration in the medium and, through the function of NCBn1, increases intracellular bicarbonate to raise pHi (5). Basal pHi in control MDCK cells was 7.36 ± 0.02, while pHi manipulation treatments significantly increased pHi to 7.49 ± 0.10 (15% CO_2_) and significantly decreased pHi to 7.17 ± 0.05 (EIPA + S0859) after 24 h (Fig. 1*A*) with no change in cell viability (Fig. S1*A*). Importantly, treatment with proteosome inhibitor MG132 (27) did not affect the magnitude of pHi changes achieved with these protocols (Fig. 1*A*). Together, these manipulation methods enable us to study how physiological changes in pHi of 0.1 to 0.3 pH units regulate β-catenin abundance and function. These pHi changes are physiologically relevant as pHi dynamics of similar magnitude occur during cellular differentiation (+0.2 pH units) (1) and cell division (±0.25 pH units) (3).

We first used Western blot approaches to assay β-catenin abundance at high and low pHi. We confirmed that high pHi decreased β-catenin abundance compared to control and found that low pHi was sufficient to stabilize β-catenin abundance compared to high pHi (Fig. 1, *B* and *C*). Importantly, we demonstrated that pH-dependent abundance of β-catenin is regulated by proteosome-mediated degradation, as treatment with proteasome inhibitor (MG132) abrogated pH-dependent abundance changes (Fig. 1, *B* and *C*). To ensure that low pHi was not inducing global protein stabilization, we probed the abundance of the AJ protein, E-cadherin (Fig. 1*D*). We found that high and low pHi conditions had no effect on the overall abundance of E-cadherin (Fig. 1*E*) or actin (Fig. S1*B*), demonstrating that the observed pH-dependent changes in β-catenin abundance are not driven by global protein degradation or stabilization. Previous work showed that phosphorylation of β-catenin was unchanged with increased pHi (5), we similarly found that phospho-β-catenin levels were not significantly different with altered pHi (Fig. S2*A*), demonstrating that protein abundance differences are not a result of pH-sensitive phosphorylation of β-catenin's degron motif. Taken together, these data suggest that pHi functions as a rheostat in regulating β-catenin abundance at the population level through a pHi- and proteasome-dependent mechanism, with increased abundance at low pHi compared to high pHi.

Because the function of β-catenin is in part determined by its subcellular localization and availability of binding partners (28, 29, 30), we next investigated how pHi alters the abundance and distribution of each subcellular pool of β-catenin. We first confirmed that the pHi manipulation techniques altered pHi in single MDCK cells using live-cell, confocal microscopy with single-cell nigericin standardization (3, 31) (Fig. S3*A*). We found that under control conditions, the median pHi value of MDCK cells was 7.39 ± 0.14 (Fig. S3*B*), in line with our previous calculations using population-level measurements (Fig. 1*A*). Importantly, treatment with EIPA + S0859 significantly lowered single-cell pHi (7.26 ± 0.17), while treatment with 15% CO_2_ significantly raised single-cell pHi (7.42 ± 0.20), relative to control MDCK cells (Fig. S3*B*).

We hypothesized that high pHi would decrease β-catenin levels in each subcellular pool, whereas low pHi would stabilize each subcellular pool. This hypothesis is predicated on recent data suggesting subcellular pools of β-catenin can rapidly exchange and that exchange is regulated by the availability of subcellular binding partners (28, 29). To test this hypothesis, we developed a 3D image analysis pipeline to segment normal epithelial cells (MDCK) and separately measure β-catenin abundance in the nucleus, cytoplasm and at AJs (Fig. S3, see methods). Briefly, cytoplasmic and nuclear regions of interest (ROIs) were generated in Nikon Imaging Software-Elements (NIS-Elements), using Hoechst dye (DNA) to identify nuclear boundaries and an E-cadherin antibody (AJs) to identify cell boundaries (Fig. S4). This segmentation was performed for every cell in the field of view, where the pixel intensity within cytoplasmic or nuclear ROIs were averaged and exported for comparison. To specifically quantify junctional pools of β-catenin, we used IMARIS (Oxford Instruments, see methods) software to generate 3D surfaces using E-cadherin intensity as a marker of AJs. This analysis pipeline enables voxel-by-voxel quantification within each E-cadherin-containing AJ surface.

**Figure 2:**
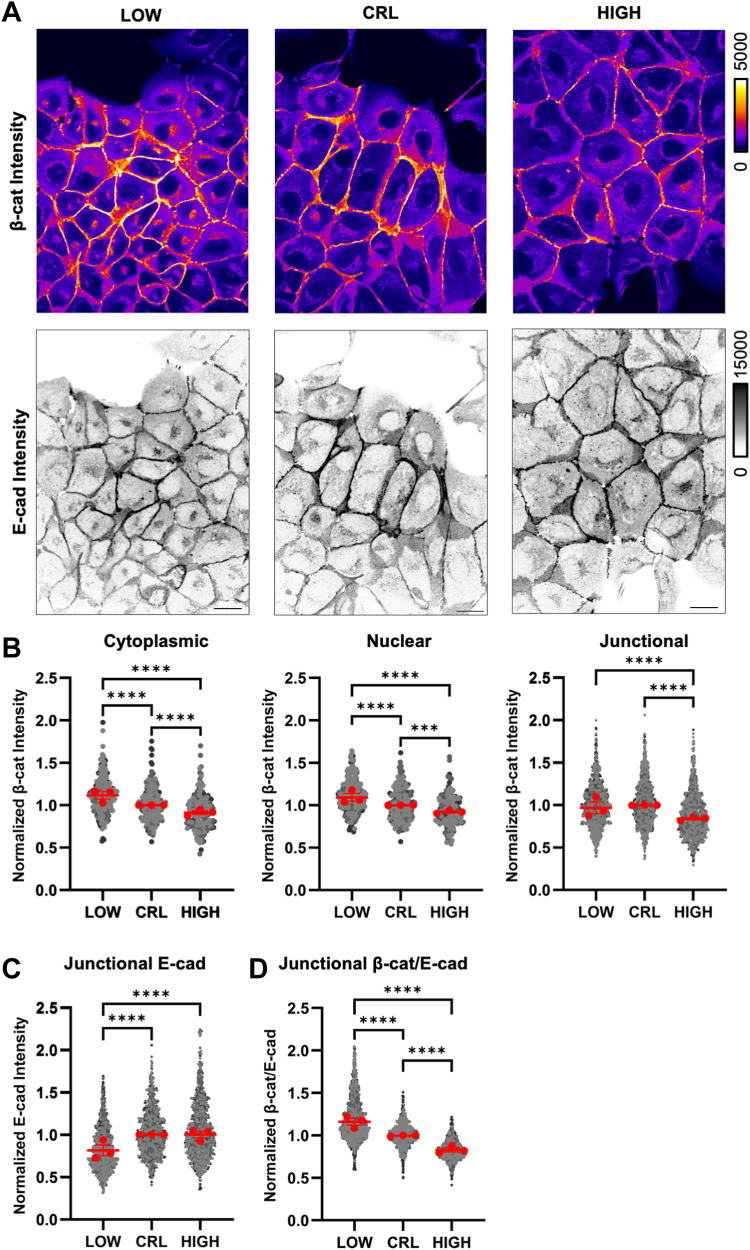
**β-catenin abundance across subcellular pools is differentially regulated by pHi**. *A*, representative confocal images of MDCK cells fixed and stained for β-catenin (β-cat) and E-cadherin (E-cad). β-catenin is pseudocolored, and E-cadherin is shown in inverse mono according to each scale. Scale bar: 20 μM. *B*, quantification of cytoplasmic, nuclear, and junctional pools of β-catenin. Individual cells and surfaces were normalized to the median of control conditions within each biological replicate. Individual cytoplasms, nuclei, and junctions are shown as *gray dots* with various shading for each biological replicate. *Red dots* represent the median for each biological replicate with interquartile ranges shown with bars. *C*, quantification of junctional E-cadherin displayed and normalized as in *B*. *D*, calculation of β-cat/E-cad ratio from quantifications in *B* and *C* that are normalized and displayed as in *B*. For *B*-*D*: data from individual cytoplasms and nuclei, n = 171 to 221; junctions, n = 934 to 1194, from three biological replicates. Statistical analyses in *B*–*D* were performed using the Kruskal–Wallis test with Dunn’s multiple comparison correction. ∗∗∗*p* < 0.001, ∗∗∗∗*p* < 0.0001. MDCK, Madin–Darby canine kidney; pHi, intracellular pH.

We first assessed the staining patterns of β-catenin and E-cadherin in MDCK cells (Fig. 2*A*). Importantly, the subcellular distribution of β-catenin in MDCK cells agrees with previous reports, with abundant membrane localization of β-catenin, weak staining of the cytoplasm, and very weak staining of nuclear β-catenin as expected for epithelial cells that are in a Wnt-inactive state (14). Using the image segmentation procedure described above, we quantified β-catenin abundance in each subcellular pool (cytoplasmic, nuclear, and junctional) after 24 h of pHi manipulation (Fig. 2*A*). We found that all subcellular pools of β-catenin decreased at high pHi (Fig. 2*B*), which agrees with the population-level Western blot data, and our hypothesis of increased pHi being sufficient to reduce β-catenin abundance across all subcellular pools. These data are also in agreement with prior quantification of β-catenin abundance at junctions and in the nucleus of MDCK cells maintained at high pHi using an alternative pH manipulation method (ammonium chloride) (5). At low pHi, we found that the cytoplasmic and nuclear pools of β-catenin were significantly increased compared to both control and high pHi conditions (Fig. 2, *A* and *B*). In our initial analysis, low pHi was not sufficient to significantly increase the abundance of β-catenin at E-cadherin-containing AJ compared to control MDCK cells (Fig. 2*B*).

To ensure we were not missing an effect, we quantified E-cadherin intensity within the AJ surfaces. Contrary to our population level measurements of total E-cadherin (Fig. 1*C*), we observed a significant decrease in E-cadherin at MDCK junctions under low pHi compared to control MDCK (Fig. 2, *A* and *C*). To control for this loss of E-cad at low pHi, we next quantified the ratio of β-catenin to E-cadherin within single MDCK junctions generated using IMARIS. We observed strong pH-dependence of β-catenin/E-cadherin ratios with increased β-catenin in E-cadherin-containing AJs at low pHi compared to control and high pHi (Fig. 2*D*). This suggests that the level of β-catenin saturation at AJs changes with pHi, with less β-catenin in E-cadherin-containing AJs at high pHi and more β-catenin in E-cadherin-containing AJs at low pHi, even though total junctional E-cadherin is reduced at low pHi.

Quantification of each pool of β-catenin provides information as to how β-catenin subcellular distribution and abundance change with respect to both increased and decreased pHi. Taken together, our data suggest that subcellular pools of β-catenin are exchangeable, with high pHi reducing cytoplasmic, nuclear, and junctional β-catenin. Similarly, our data suggest low pHi results in cytoplasmic and nuclear accumulation of β-catenin and a relative increase in β-catenin at E-cadherin-containing AJs.

As we were analyzing these data, we noted a distinct change in cell morphology of the fixed cells across our treatment conditions, with apparent pH-dependent cell size changes (Fig. 2*A*). To quantify these changes in live cells, we used CellMask DeepRed to label the plasma membranes of live MDCK cells under low, control, and high pHi conditions and collected 3D confocal image stacks of the cells to quantify both cell cross-sectional area and volume (Fig. S5*A*). While cell volume was unchanged across pHi treatment conditions (Fig. S5*B*), cell cross-sectional area decreased at low pHi compared to control MDCK and increased at high pHi compared to control (Fig. S5*C*). Prior work has shown that cell cross-sectional area increases when cells have higher cell-matrix adhesion, while cell cross-sectional area is smaller in cells with high cell–cell adhesion (32). Thus, our data demonstrate that pH-dependent changes in junctional β-catenin can drive cell shape changes that are associated with altered cell–cell adhesion. We next probed whether AJ composition was altered by pHi dynamics.

### Endogenous plakoglobin compensates for pHi-dependent loss of β-catenin to maintain AJs at high pHi

In the immunofluorescence assays of β-catenin, we observed no change in MDCK colony formation with altered pHi, even when junctional pools of β-catenin were significantly decreased at high pHi (Fig. 2, *A* and *B*), and cell cross-sectional area was increased (Fig. S5). This suggests that other AJ proteins may be able to rescue the loss of junctional β-catenin to maintain AJ integrity. While β-catenin plays a crucial role in maintaining AJs in epithelial cells, plakoglobin serves adhesive functions in both AJs and desmosomes, with preferential incorporation into the latter (33). Work by Fukunaga *et al*. demonstrated the functional redundancy of β-catenin and plakoglobin in maintaining AJ integrity, where cell–cell contacts can be rescued with plakoglobin expression in a β-catenin null background (34). Furthermore, knockout of β-catenin in mouse models resulted in significant recruitment of plakoglobin to AJs, compensating for the loss of AJ-associated β-catenin (35). A similar effect was observed in mouse models using conditional β-catenin knockdown in cardiomyocytes, where β-catenin was reduced by 90%, and plakoglobin was subsequently enriched at junctions (36). We therefore hypothesized that in MDCK cells, the homologous protein plakoglobin can rescue β-catenin loss from AJs at high pHi to preserve MDCK epithelial morphology.

**Figure 3:**
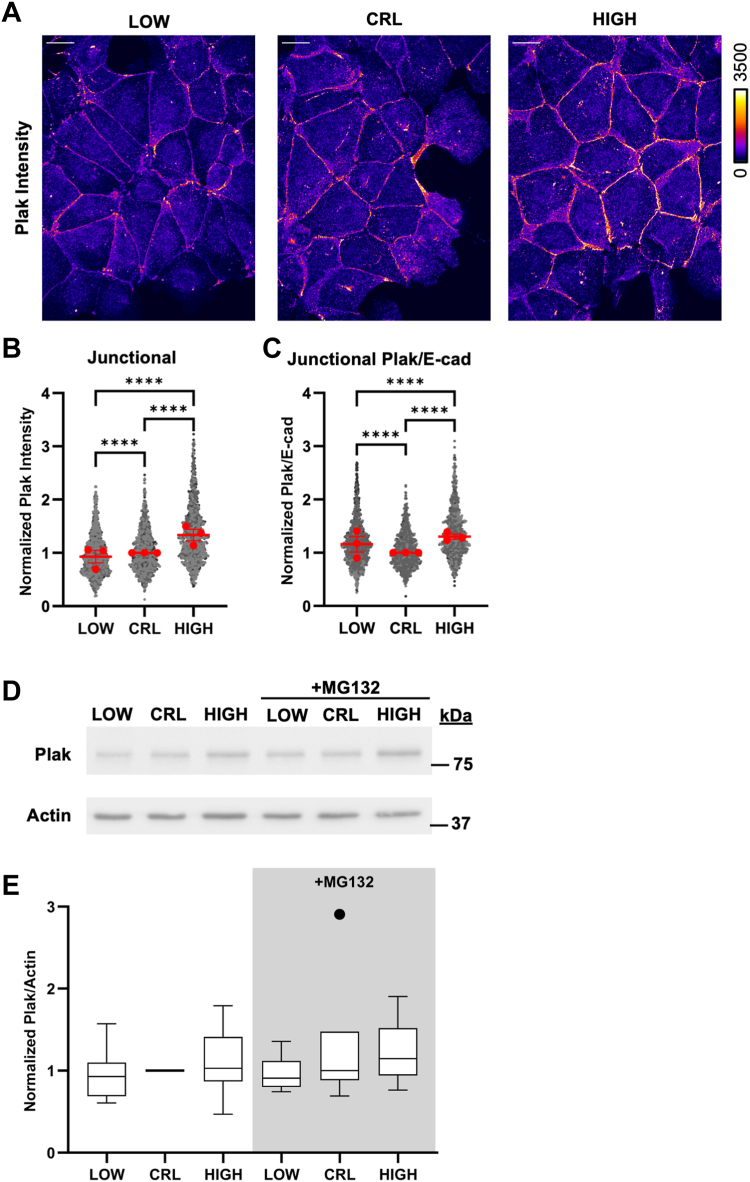
**Endogenous plakoglobin compensates for loss of β-catenin from junctions at high pHi**. *A*, representative confocal images of MDCK cells fixed and stained for Plakoglobin (Plak). Plakoglobin is pseudocolored according to scale. Scale bar: 20 μM. *B*, *C*, quantification of junctional pools of Plakoglobin (*B*) and Plak/E-cad ratios (*C*) from individual surfaces. Individual surfaces were normalized to the median of control conditions within each biological replicate. Individual junctions are shown as *gray dots* with various shading for each biological replicate, n = 934 to 1194, from three biological replicates. *Red dots* represent the median for each biological replicate with interquartile ranges shown with bars. *D*, representative immunoblot of Plak and actin under low, control, and high pHi conditions in the presence and absence of proteosome inhibition (MG132). *E*, quantification of Plak immunoblot data collected as described in *D*. Individual biological replicates were normalized to control MDCK within each experiment. *Box* and *whisker* plots show median (*line*), 25th-75th percentile (*boxes*), min and max (*whiskers*), and outlier values with points; from five to six biological replicates. Statistical analyses of *B* and *D* were performed using the Kruskal–Wallis test with Dunn’s multiple comparison correction. Statistical analyses of *C* and *E* were determined with ratio paired *t* tests between treatment groups and one-sample t-tests with a hypothetical mean of 1.0 when comparing to control (which had no variation due to normalization). ∗∗∗∗*p* < 0.0001. MDCK, Madin–Darby canine kidney; pHi, intracellular pH.

To determine whether plakoglobin compensates for the loss of β-catenin at AJs at high pHi, we performed immunofluorescent confocal microscopy of plakoglobin using the same segmentation pipeline (outlined in Fig. S4) after 24 h of pHi manipulation. We found that junctional-associated plakoglobin significantly increased at high pHi and significantly decreased at low pHi compared to control MDCK (Fig. 3, *A* and *B*, C). Importantly, overall abundance of plakoglobin is unchanged by pHi (Fig. 3, *D* and *E*) suggesting a change in localization is driving these observations. These data, combined with the analysis of β-catenin abundance at E-cadherin-containing junctions (Fig. 2), do suggest that pHi dynamics can remodel AJ composition. However, the ratioed voxel-by-voxel analyses are still limited as we are comparing median ratioed intensity values that are then normalized from one replicate to the next. We wanted to determine functional correlations between expression level of one AJ protein and another in our data.

Thus, to characterize AJ composition in more detail, we generated voxel-by-voxel correlation plots of normalized β-catenin and plakoglobin intensities *versus* E-cadherin intensities in E-cadherin containing AJs. Importantly, because we performed simultaneous labeling of E-cadherin, plakoglobin, and β-catenin, these correlation maps are robust across biological replicates. As expected, we found that both β-catenin and plakoglobin intensities were positively correlated with E-cadherin intensity (Fig. S6, *A* and *B*). When we analyzed the correlation plots of β-catenin, we found a significant increase in β-catenin abundance at low pHi compared to high pHi, as evidenced by the distinct separation of scatter plot data by pHi [shifted along the y axis (β-catenin intensity)] (Fig. S6*A*). When we analyzed the correlation plots of plakoglobin *versus* E-cadherin, we found no change in overall plakoglobin abundance, as evidenced by the overlap of scatter plot data by pHi (Fig. S6*B*).

**Figure 4:**
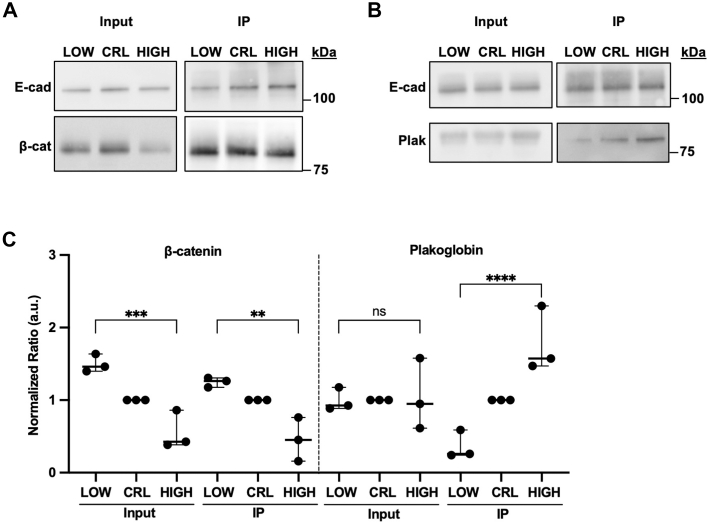
**Immunoprecipitation of E-cadherin shows AJ composition is altered with changes in pHi**. *A*, representative immunoblots of plakoglobin (Plak) and E-Cadherin in whole cell lysates (*left*, input) and in E-cadherin containing immune complexes (*right*, IP). *B*, representative immunoblots of β-catenin (β-cat) and E-cadherin in whole cell lysates (*left*, input) and in E-cadherin containing immune complexes (*right*, IP). *C*, quantification of plakoglobin and β-catenin in E-cadherin immunocomplexes collected as described in *A*. Individual biological replicates were normalized to control MDCK within each experiment. Scatter plots show all data points from n = 3 biological replicates. Statistical significance was determined using a one-way ANOVA between high and low treatment groups. ∗∗*p* < 0.01, ∗∗∗*p* < 0.001, ∗∗∗∗*p* < 0.0001, ns ,not significant; AJ, adherens junction; MDCK, Madin–Darby canine kidney; pHi, intracellular pH.

Taken together, these correlation data recapitulate the pH-dependent abundance data showing β-catenin has pHi-dependent abundance (Fig. 2) while plakoglobin does not (Fig. 4). These scatter plots additionally show that low pHi uniformly increases β-catenin abundance in E-cadherin-containing voxels independently of E-cadherin concentration. In support of this, we found correlation coefficients between E-cadherin and β-catenin were unchanged with changing pHi (Fig. S6*C*). This suggests that the observed β-catenin abundance changes at AJs (Fig. 2 and Fig. S6*A*) are not being driven by changes in β-catenin affinity for E-cadherin (which would be dependent on the concentration of E-cadherin and thus affect correlations) but instead are driven by overall changes in β-catenin abundance (which would increase binding independently of E-cadherin concentration). Importantly, we found that β-catenin had higher correlation with E-cadherin at low pHi (Spearman r = 0.71) compared to plakoglobin (Spearman r = 0.43, *p* < 0.0001) (Fig. S6, *C* and *D*). We also found that plakoglobin was more highly correlated with E-cadherin at high pHi (Spearman r = 0.55) compared to low pHi (Spearman r = 0.43; *p* < 0.0001) (Fig. S6, *C* and *D*), suggesting plakoglobin abundance is not being directly regulated and instead it is being relocalized to AJs at high pHi in a concentration-dependent manner.

We next biochemically confirmed that pHi dynamics alter the composition of E-cadherin-based AJs. We prepared lysates from MDCK cells under low, control, or high pHi conditions and performed immunoprecipitation of E-cadherin. We found that β-catenin was enriched in E-cadherin immune complexes at low pHi compared to control and high pHi (Fig. 4, *A* and *C*). Conversely, plakoglobin was enriched in E-cadherin immune complexes at high pHi compared to control and low pHi (Fig. 4, *B* and *C*). This result confirms the pH-dependent colocalization and correlation data shown in single cells (Figure 2, Figure 3, and Fig. S6). In summary, our data show that while β-catenin colocalization with E-cadherin is reduced at high pHi compared to control (Fig. 2*D* and 4*B*), plakoglobin colocalization with E-cadherin is increased (Fig. 3*C* and 4*C*). While further work is required to fully characterize pH-dependent AJ remodeling, our data suggest that when β-catenin is lost from junctions at high pHi, plakoglobin is recruited to stabilize AJs. Similarly, when β-catenin is stabilized at E-cadherin-containing AJs at low pHi, plakoglobin abundance in AJs is reduced.

### Characterization of pH-dependent β-catenin degradation

Prior work has already extensively examined roles for high pHi in reducing β-catenin stability (5), but the effects of low pHi on β-catenin stability have not been characterized. We next measured the temporal localization and stability dynamics of endogenous β-catenin at the population level, with the prediction that low pHi increases β-catenin stability resulting in the observed increased β-catenin abundance at low pHi. We first quantified pH-dependent stability of β-catenin by performing a cycloheximide chase assay (37) after 24 h of pHi manipulation in MDCK cells, collecting samples every 30 min for 2.5 h (Fig. S7*A*). The half-life of endogenous β-catenin under control condition was determined to be 1.58 h, in agreement with prior work in MDCK cells (5). Importantly, we found that β-catenin half-life was increased at low pHi (2.79 h) and decreased at high pHi (1.22 h) compared to control (Fig. S7*B*). Of note, White *et al*. used different treatment conditions to raise pHi (ammonium chloride compared to our method of 15% CO_2_) (5). Thus, our work indicates that high pHi accelerates β-catenin degradation, regardless of the method of pHi manipulation. Finally, this work is the first to report an extension of β-catenin half-life in cells under low pHi conditions.

### An experimental approach to monitor β-catenin degradation rates in single cells

The population-level analyses of β-catenin stability using cycloheximide demonstrated pH-dependent stability, where low pHi increased and high pHi decreased β-catenin stability (half-life) relative to control. Although these data revealed altered rates of β-catenin degradation with pHi manipulation, these population-level analyses require discontinuous measurements across cell populations and lack both single-cell and temporal resolution. Single-cell analyses are important to better connect and support emerging roles for pH-dependent β-catenin regulation in processes that occur at the single-cell level, such as differentiation (1). One approach for quantifying live- and single-cell protein dynamics is to monitor a photoconvertible fluorescent protein (PCFP) fused to the protein of interest. PCFPs are a subfamily of fluorescent proteins that undergo an excitation and emission spectra shift upon stimulation with a specific wavelength of light (38). This approach enables the photoconversion of a small subpopulation of the PCFP-tagged protein, allowing for specific tracking of the photoconverted protein fluorescence intensity as a direct readout of both protein localization changes and degradation (after accounting for photobleaching effects). This approach also eliminates the need to average bulk cell responses as with cycloheximide chase assays, enabling investigation of how pHi regulates protein degradation in single cells.

To monitor live-cell β-catenin protein degradation rates, we selected mMaple3, a PCFP that transitions from green-to-red fluorescence upon exposure to 405 nm light (39). Derived from the parent protein mMaple, mMaple3 has reduced dimerization tendency, superior brightness in the converted channel, and improved photoconversion efficiency (defined as the proportion of protein that yields detectable fluorescence signal) (39, 40, 41). To validate that we can measure differences in protein degradation rates of photoconverted mMaple3 using confocal microscopy, we transiently expressed mMaple3 alone or N-terminally fused to β-catenin (mMaple3-β-catenin) or a degradation resistant β-catenin mutant (H36R-β-catenin) (5).

**Figure 5:**
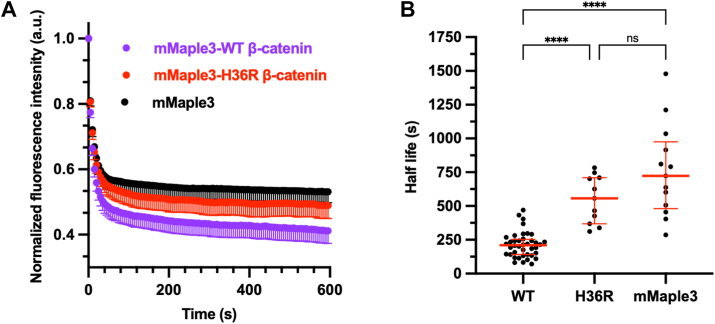
**mMaple3 photoconversion can report on relative single-cell protein degradation rates**. *A*, normalized fluorescence intensity after photoconversion of mMaple3-β-catenin, mMaple3-H36R-β-catenin, and mMaple3 alone. Shown are mean cell traces, and error bars below mean indicate SEM. *B*, protein half-life was calculated or each cell in *A* and shown in a scatter plot (median ± IQR) (mMaple3, n = 12 single cells, N = 3 biological replicates; mMaple3-H36R-β-catenin, n = 11 single cells, N = 3 biological replicates; mMaple3-β-catenin, n = 19 single cells, N = 4 biological replicates). Statistical significance in *B* was determined using Kruskal–Wallis test with Dunn’s correction for multiple comparisons. ∗∗∗∗*p* < 0.0001, ns, not significant.

We hypothesized that this approach would allow for comparisons of relative protein degradation rates in single cells across treatment conditions. We first tested whether we could observe protein half-life differences in MDCK cells expressing mMaple3 alone, wildtype β-catenin, or a degradation-resistant β-catenin mutant (H36R-β-catenin). We photoconverted the target protein using a spatially restricted region of interest (Video 1, β-catenin). We calculated normalized mean fluorescence intensity decay curves from cells and found that wildtype β-catenin was lost more rapidly than either H36R-β-catenin or mMaple3 alone (Fig. 5*A*). It is important to note that this assay does not permit the determination of absolute protein half-life, as loss of fluorescent signal in this assay includes protein degradation, diffusion, and photobleaching of the PCFP. Indeed, we found that all proteins (including mMaple3) exhibited a two-phase fluorescence decay curve with a rapid decay followed by a slower loss of signal (Fig. 5*A*). All subsequent analyses used two-phase decay curve fits and reported half-life values are for the slow rate, as the fast rates were not different across the proteins studied and likely reflect effects of diffusion.

We next determined protein half-life from single-cell normalized fluorescence decay curves. Using this approach, we found that the median single-cell half-life of mMaple3 was 722 s, while the median single-cell half-life of mMaple3-β-catenin was significantly reduced to just 209 s (Fig. 5*B*). This suggests that the shorter mMaple3-β-catenin half-life is being driven by ubiquitination and degradation of β-catenin in the fusion protein. Confirming this, a degradation-resistant mutant of β-catenin (mMaple3-H36R-β-catenin) had a significantly increased median half-life of 557 s in this assay, which was not statistically significantly different from that of mMaple3 alone (Fig. 5, *A* and *B*). Taken together, these data demonstrate mMaple3-β-catenin is (1) degraded much faster than mMaple3 alone and (2) can be stabilized using a degradation-resistant mutant (H36R). Thus, these data validate the single-cell system as a reporter of mMaple3-β-catenin degradation dynamics in single cells and allow us to compare relative half-life values between pHi conditions. Next, we measured mMaple3-β-catenin degradation rates in single MDCK cells with pHi manipulation.

### pHi titrates the degradation rates of β-catenin in single epithelial cells

**Figure 6:**
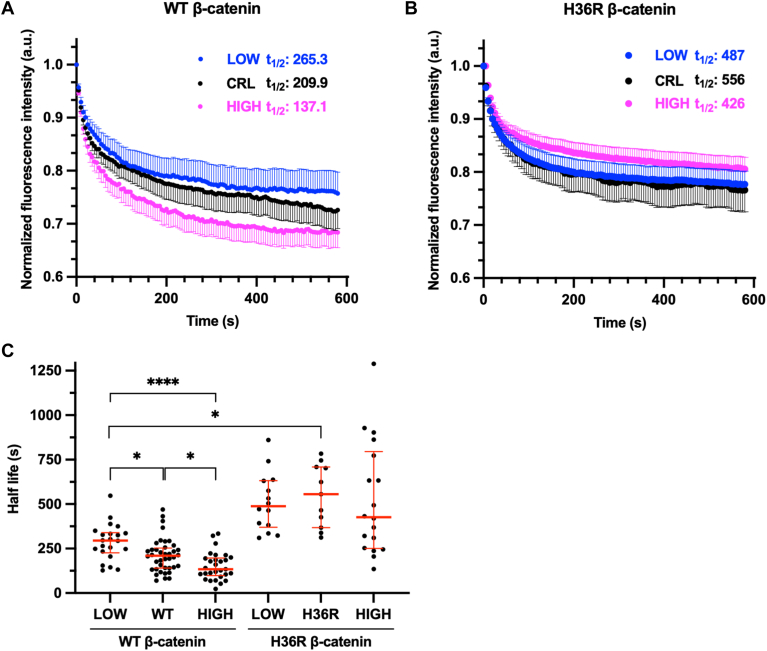
**mMaple3-β-catenin has pH-dependent degradation, while mMaple3-H36R-β-catenin is stabilized and pH-insensitive**. *A*, normalized fluorescence intensity traces from MDCK cells expressing mMaple3-β-catenin under low, control, and high pHi conditions. *Dots* show mean cell traces, and error bars indicate SEM. Reported protein half-life calculated from mean cell traces. LOW, n = 14; CRL, n = 9; HIGH, n = 20 from three biological replicates. *B*, normalized fluorescence intensity traces from MDCK cells expressing mMaple3-β-catenin under low, control, and high pHi conditions. *Dots* show mean cell traces, and error bars below (CRL) or above (HIGH, LOW) mean indicate SEM. Reported protein half-life calculated from mean cell traces. LOW, n = 14; CRL, n = 11; HIGH, n = 18 from three biological replicates. *C*, single-cell protein half-life values were obtained by fitting individual single-cell traces from cells collected as in *A* and *B*. (median ± IQR) Outliers were removed using the ROUT method (Q = 1%). WT LOW, n = 22; WT CRL, n = 40; WT HIGH, n = 29 from six biological replicates. H36R LOW, n = 14; H36R CRL, n = 11; H36R HIGH, n = 18 from three biological replicates. Statistical significance in *C* was determined using Kruskal–Wallis test with Dunn’s correction for multiple comparisons. ∗*p* < 0.05, ∗∗∗∗*p* < 0.0001. MDCK, Madin–Darby canine kidney.

We altered pHi in mMaple3-β-catenin-expressing MDCK cells and photoconverted cytoplasmic mMaple3-β-catenin, enabling us to monitor relative pH-dependent degradation rates of cytoplasmic β-catenin in single cells. We observed a reduced mean half-life at high pHi (137.1s) and increased mean half-life at low pHi (265.3s) compared to control MDCK cells (209.9s) (Fig. 6*A*). Our photoconversion experiments enable fitting of single-cell half-life curves, which similarly showed that low pHi significantly increased median mMaple3-β-catenin single-cell half-life compared to control and high pHi conditions (Fig. 6*C*). We performed the same photoconversion experiments using the stabilized β-catenin mutant (H36R-β-catenin) and found that the mean half-life of mMaple3-H36R-β-catenin was increased across all pHi conditions compared to mMaple3-β-catenin (Fig. 6*B*), suggesting that turnover of WT β-catenin is faster than the stabilized mutant under all pHi conditions. Importantly, median single-cell half-life of mMaple3-H36R-β-catenin was also pHi-insensitive across all conditions tested (Fig. 6*C*). This confirms prior work showing His36 is essential for pHi-dependent β-catenin stability (5). As a control, we also assessed mMaple3 stability in MDCK cells with and without pHi manipulation and found no difference in mean half-life or median single-cell half-life for mMaple3 (Fig. S8). These experiments demonstrate that pHi titrates the degradation rate of cytoplasmic β-catenin in single cells, with faster degradation at high pHi and slower degradation at low pHi compared to control. These results also confirm that His36 is critical for pHi-dependent stability of β-catenin in single living cells.

**Figure 7:**
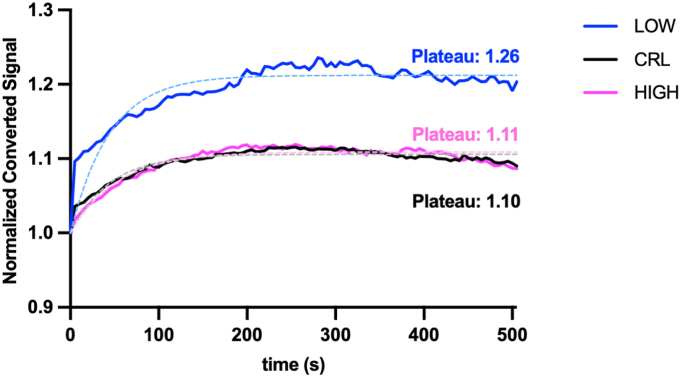
**Low pHi increases nuclear accumulation of cytoplasmic photoconverted mMaple3-β-catenin**. Normalized median cell traces for each condition are plotted and were fit using nonlinear regression (fitted curves shown as dashed, *tinted lines*) to obtain plateau values. LOW, n = 14; CRL, n = 13; HIGH, n = 11; from 3 biological replicates. Statistical comparison was performed by determining whether a single curve could adequately fit both datasets, the null hypothesis was rejected (*p* < 0.0001). pHi, intracellular pH.

In the photoconversion experiments described above, we performed cytoplasmic photoconversion and specific tracking of the cytoplasmic pool. This approach also allows us to monitor subcellular redistribution of photoconverted cytoplasmic mMaple3-β-catenin into the nucleus under altered pHi conditions. We measured nuclear accumulation of photoconverted mMaple3-β-catenin following cytoplasmic photoconversion and observed significantly increased nuclear accumulation of mMaple3-β-catenin at low pHi compared to high pHi cells (Fig. 7). These data suggest that pHi modulates nuclear accumulation of photoconverted β-catenin and supports our previous observation that nuclear β-catenin abundance is increased at low pHi compared to control and high pHi (Fig. 2*B*).

Our work suggests that low pHi significantly increases β-catenin abundance in the cytoplasm and nucleus due to attenuated cytoplasmic degradation. A remaining unanswered question is whether the transcriptional activity of wildtype, endogenous β-catenin is altered as a consequence of pH-dependent degradation of cytoplasmic protein pools, as this was not addressed in prior work investigating pH-dependent β-catenin stability (5). To address this, we tested the hypothesis that low pHi increases the transcriptional activity of endogenous β-catenin, whereas high pHi reduces transcriptional activity.

### Decreased pHi is sufficient to increase transcriptional activity of β-catenin

**Figure 8:**
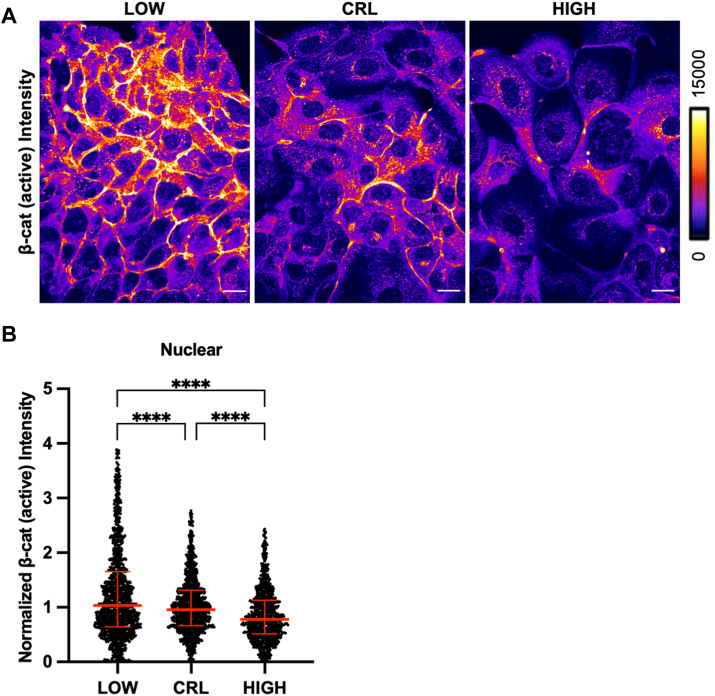
**Active β-catenin is increased in the nucleus at low pHi compared to control and high pHi**. *A*, representative confocal images of MDCK cells fixed and stained for nonphosphorylated (active) β-catenin (Ser33/Ser37/Thr41). β-catenin is pseudocolored according to scale. Scale bar: 20 μM. *B*, quantification of nuclear intensities, normalized to the median of control conditions within each biological replicate. Individual cells are shown as *gray dots*, (n = 756–1231, from four biological replicates). Medians and interquartile ranges shown in *red*. Statistical significance for *B* and *C* as determined by Kruskal–Wallis test with Dunn’s multiple comparisons correction. ∗∗∗∗*p* < 0.0001. pHi, intracellular pH.

While the function of β-catenin is regulated by its localization, recent work revealed that nuclear localization of total protein alone is not sufficient to determine β-catenin function (42). Thus, to further support these data, we assessed nuclear abundance of nonphosphorylated (transcriptionally active) β-catenin in single cells with altered pHi. As expected, immunofluorescence staining for active β-catenin (Fig. 8*A*) showed that active β-catenin enriched at cell membranes and is increased in the nucleus of MDCK cells at low pHi compared to control and high pHi cells (Fig. 8*B*). These data demonstrate that low pHi increases transcriptionally active forms of β-catenin in the nucleus, suggesting that pHi-dependent regulation of β-catenin abundance may also regulate transcriptional activity.

**Figure 9:**
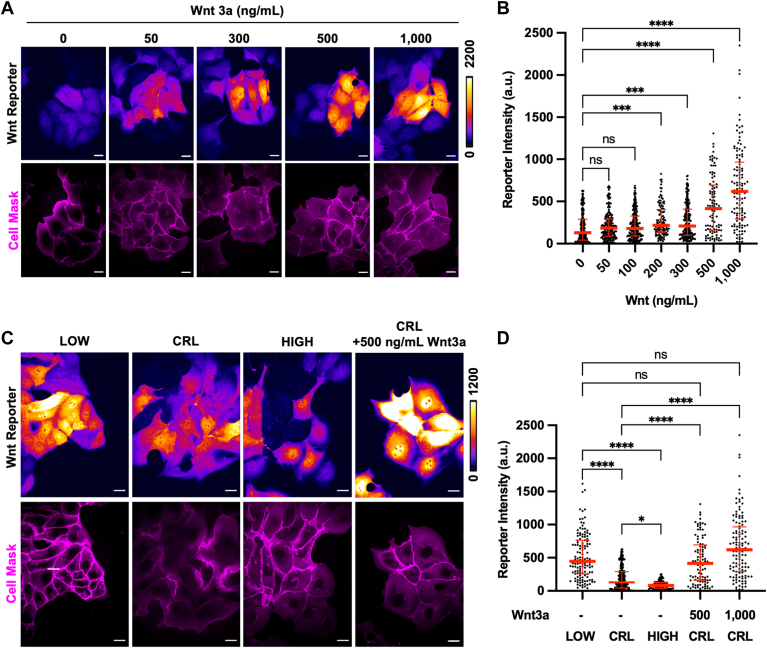
**Low pHi phenocopies Wnt3a-mediated activation of β-catenin transcription**. *A*, representative images of control MDCK cells expressing a TOPdGFP reporter with cell membranes visualized with Cell Mask (*magenta*). Cells were treated with Wnt3a at the indicated concentrations (0–1000 ng/ml) for 24 h. Scale bar = 20 μm. *B*, quantification of single-cell reporter intensities from cells prepared as in A. Individual cells are shown as *block dots* with median and interquartile ranges shown in *red lines*; n = 97 to 240 cells across three biological replicates. *C*, representative images of MDCK cells expressing a TOPdGFP reporter plasmid with and without pHi manipulation. Intensity shown according to scale, with cell membranes visualized with Cell Mask (*magenta*). *D*, quantification of single-cell reporter intensities from cells prepared as in C. Individual cells are shown as *block dots* with median and interquartile ranges shown in *red lines*; n = 86 to 240 cells across three biological replicates. For *B* and *D*, statistical significance was determined using Kruskal–Wallis test with Dunn’s correction for multiple comparisons. ∗*p* < 0.05, ∗∗∗*p* < 0.001, ∗∗∗∗*p* < 0.0001. MDCK, Madin–Darby canine kidney; pHi, intracellular pH.

To test this hypothesis, we next performed single-cell analysis of pH-dependent β-catenin transcriptional activity using a highly specific LEF-1/TCF transcriptional reporter plasmid that drives expression of destabilized GFP (TOPdGFP) (43). We generated a stable clonal MDCK cell line expressing this reporter (see methods). We first validated that the reporter cell line responded to Wnt3a stimulation across a range of Wnt3a concentrations (Fig. 9, *A* and *B*). We then manipulated pHi for 24 h prior to live-cell imaging of reporter fluorescence using confocal microscopy (Fig. 9*C*). We observed a statistically significant increase in reporter fluorescence at low pHi compared to high pHi (Fig. 9*D*). Importantly, lowering pHi produced reporter fluorescence that was statistically indistinguishable from the highest concentrations of Wnt3a tested (Fig. 9*D*). We also found that high pHi reduced transcriptional activation of β-catenin compared to control (Fig. 9, *C* and *D*). This demonstrates that high pHi can reduce basal β-catenin transcriptional activity, even in the absence of Wnt-3a stimulation. These single-cell analyses show that low pHi alone can recapitulate the Wnt-active state in MDCK cells, validating low pHi as a sufficient driver of endogenous β-catenin transcriptional activity in normal epithelial cells.

### Structural support for pH-sensitive β-catenin degradation

A high-resolution crystal structure of β-TrCP bound to doubly phosphorylated (pSer33/pSer37) β-catenin (1P22) (44) was previously used to support a hypothesis for increased β-TrCP binding and degradation of β-catenin at high pHi (5). However, more recent work has produced two relevant crystal structures that enable a more comprehensive analysis of β-catenin binding poses on the β-TrCP WD40 repeats. Simonetta *et al*. collected structures of β-TrCP bound to singly phosphorylated (pSer33) β-catenin (6M94) (45), which has reduced binding compared to doubly phosphorylated (pSer33/pSer37) β-catenin (1P22). We compared these two structures and found that the His36 binding pose is very different, with His36 flipped inward toward β-TrCP in the high-affinity structure (1P22), interacting with both Lys365 and the carbonyl backbone of pSer33, while the low-affinity structure (6M94) shows His36 flipped outward and not interacting with Lys365 or the carbonyl backbone of pSer33 (Fig. S9, *A* and *B*). Metrics comparisons of the two structures show that in the higher affinity (doubly phosphorylated β-catenin, 1P22) structure, His36 is more buried (58% *versus* 23%), has a lower predicted pKa (4.6 *versus* 5.79), and a stronger coulombic interaction with Lys365 of β-TrCP (−0.45 *versus* −0.36) with shorter measured His36-Lys365 heteroatom distances (4.0Å *versus* 4.3Å) compared to the lower affinity structure (6M94) (Fig. S9*F*).

The study by Simonetta *et al*. also produced a crystal structure of a ternary complex of β-TrCP, singly phosphorylated (pSer33/Ser37) β-catenin, and a small molecule that enhances degradation of the singly phosphorylated mutant by over 100-fold (6M90). This provides another opportunity for comparing a validated high affinity structure (6M90) and low affinity structure (6M94). We compared 6M90 and 6M94 and found again that the His36 of β-catenin flipped inward toward β-TrCP in the high-affinity structure (6M90), interacting with Lys365 of β-TrCP and the carbonyl backbone of pSer33 while the low-affinity structure shows His36 flipped outward and not interacting with the carbonyl backbone of pSer33 (Fig. S9, *B* and *C*). Again, metrics comparisons of the two structures show that in the higher affinity (6M90) structure, His36 is more buried (59% *versus* 23%), has a lower predicted pKa (5.26 *versus* 5.79) and a stronger coulombic interaction with Lys365 of β-TrCP (−0.39 *versus* −0.36) but has similar measured His36-Lys365 heteroatom distances (4.7Å *versus* 4.3Å) compared to the lower affinity structure (6M94) (Fig. S9*F*).

One important caveat to the data above is that all structures were collected from crystals generated at low buffer pH (5.5–6.8), which we have shown reduces the affinity of β-catenin for β-TrCP *in vitro* (5). Thus, we expect that these structures still represent a moderate binding affinity compared to the highest binding affinity achieveable at high pH values. Because of this, we also generated AlphaFold structures of doubly phosphorylated (pSer33/pSer37) β-catenin and β-TrCP. While AlphaFold does not allow explicit pH tuning, protonation states during structure calculation reflect a pH environment of 7.2 (46). As expected, we found the AlphaFold structures depicted His36 of β-catenin in the “flipped in” conformation, interacting with Lys365 of β-TrCP and the carbonyl backbone of pSer33, overlapping with the 6M94 high affinity structure (Fig. S9, *B* and *C*). We did notice that the high affinity, high pH (AlphaFold) structure has a His36 pose that is more buried (85% *versus* 59%), has a lower predicted pKa (4.7 *versus* 5.26), and a stronger coulombic interaction with Lys365 of β-TrCP (−0.49 *versus* −0.39) when compared to the lower affinity, low pH structure (6M94) (Fig. S9, *D* and *F*).

While future experimental work will expand on the molecular characterization of the pH-dependent β-catenin phosphodegron-binding activity of β-TrCP, these computational analyses suggest that in high-affinity binding poses, His36 of β-catenin is deprotonated, enabling buried R group and favorable coulombic interactions with Lys365. This is supported by the pKa predictions we made based on available crystal structures and PROPKA (Fig. S9*F*). The pKa of His36 in the low-affinity binding structures is higher (indicating an increased likelihood of being protonated at physiological pH) compared to the high-affinity binding structures (indicating an increased likelihood of being deprotonated at physiological pH).

### Evolutionary conservation of histidine-containing phosphodegrons

The phosphodegron of β-catenin (DSGI**H**S) is highly evolutionarily conserved, but there is some variation in this motif, with instances of DSGI**N**S in some insects; DSGI**Q**S in worms, lampreys, and marine animals; and DSGI**V**S in some fish and sea cucumbers (Fig. S10, Table S1). Thus, pH-dependent regulation of stability mediated by His36 may vary across species, suggesting that pH-dependent regulation of Wnt signaling may not be a uniform response. Furthermore, there are other substrates of β-TrCP that have a (DSGI**H**S) motif, including plakoglobin, though the evolutionary conservation of His at this position of the motif is not as strong as that observed for β-catenin, with Q being more frequently found in this position in plakoglobin (Fig. S10, *B* and *C*). Importantly, we did not observe pH-dependent degradation of plakoglobin, indicating that the presence of histidine in the phosphodegron motif, which is present in both β-catenin and plakoglobin, is not a sufficient indicator of resulting pH-dependent stability. There could be many underlying reasons why plakoglobin does not exhibit pH-dependent stability even though it contains a histidine in the phosphodegron motif. We hypothesize that desmosome localization of plakoglobin may protect it from global stability effects in the context of the cell. There is literature precedence for this with several groups showing slow exchange of desmosome-localized proteins with the cytoplasm (47, 48). To test this hypothesis, future work will test pH-dependence of β-TrCP and plakoglobin binding affinity *in vitro* and compare it to our cell-based data. An alternative hypothesis is that plakoglobin binding pose is different across the β-TrCP WD40 repeats, perhaps avoiding β-TrCP-Lys365 coulombic interactions that we predict are driving pH-dependent β-catenin degradation. These “alternate binding poses” compared to what is observed in β-catenin structures have been proposed for some β-TrCP targets (49, 50) using molecular dynamics simulations and have been shown in the literature for nonhistidine-containing β-TrCP targets (51). Thus, we conclude that phosphodegrons containing a histidine in the primary sequence may indicate the possibility of pH-dependent degradation, but our work shows primary sequence analysis is not sufficient to identify pH-sensitive β-TrCP substrates. Future experimental work will expand on the molecular characterization of pH-dependent phosphodegron-binding activity across known β-TrCP targets.

## Discussion

Our data highlight an important role for pHi in serving as a rheostat to regulate β-catenin abundance, where low pHi increases β-catenin stability, which increases β-catenin colocalization to E-cadherin and drives increased transcriptional activity even in the absence of extracellular Wnt signal. Conversely, high pHi reduces β-catenin stability, which reduces β-catenin at E-cadherin-containing AJs and lowers β-catenin transcriptional activity compared to control cells. Our work significantly expands on prior work that only explored the effects of increased pHi on β-catenin abundance and did not fully characterize pH-dependent regulation of β-catenin function. Our work demonstrates that pHi changes within the physiological range can regulate the degradation, abundance, and function of endogenous β-catenin.

We also found that subcellular pools of β-catenin were differentially regulated by pHi. We show that pHi-mediated loss of β-catenin from E-cadherin-containing AJs can be rescued by plakoglobin to maintain AJ integrity at high pHi. While genetic knockdown of β-catenin was previously shown to recruit plakoglobin to AJs (34), we demonstrate that increased degradation of β-catenin at high pHi is sufficient to produce the same compensatory phenotype at AJs. We also show that at low pHi, β-catenin is increased, and plakoglobin is reduced at E-cadherin-containing AJs. Thus, the composition of AJs is altered by pHi dynamics, with a preference for β-catenin at low pHi and a preference for plakoglobin at high pHi. This presents a potential novel mechanism of pH-dependent AJ remodeling that may be a previously unrecognized reinforcer of pH-dependent cell polarity (52) or EMT (53). Moreover, the increased ratio of plakoglobin to E-cadherin at high pHi may also be indicative of downstream changes in desmosome formation at high pHi, as plakoglobin-containing AJs are a prerequisite for their establishment (47). Future work will explore the molecular mechanisms of pH-dependent AJ remodeling, characterize how the physical properties of AJ change with pHi, and expand on the functional output of this remodeling in the context of 3D tissue formation.

Collectively, our single-cell level analyses of β-catenin transcriptional activity demonstrate that modest stabilization of nuclear β-catenin is sufficient to drive significant changes in transcriptional activity. We found that low pHi increased β-catenin transcriptional activity and phenocopied transcriptional outcomes of Wnt3a treatment. Importantly, while plakoglobin can rescue the AJ function of β-catenin at high pHi, our data suggest plakoglobin cannot rescue the reduced signaling function of β-catenin at high pHi, as transcriptional activity at high pHi was significantly reduced compared to control. This aligns with prior work showing β-catenin/TCF4 complexes are transcriptionally active but that plakoglobin/TCF4 complexes are unable to bind DNA (54, 55). Our data show that pHi directly regulates β-catenin signaling and transcriptional activity in the absence of Wnt pathway activation and indicates that pH-dependent regulation of β-catenin signaling may indeed reinforce stem cell phenotypes previously associated with low pHi (4) and high Wnt signaling (56).

Others have shown that internalization of β-catenin: E-cadherin complexes upon growth factor stimulation causes increased transcriptional activity of β-catenin, suggesting β-catenin: E-cadherin complex formation is a prerequisite for signaling-competent β-catenin (57). Here, we observed that low pHi induced (1) an unexpected loss of junctional E-cadherin, (2) increased β-catenin in E-cadherin immune complexes from whole cell lysates, and (3) increased transcriptional activity of β-catenin. Our data may suggest that low pHi enhances the internalization of E-cadherin saturated with β-catenin which may result in more transcriptionally active β-catenin relocalizing to the nucleus. While not directly tested in this work, synergy between enhanced β-catenin/E-cadherin internalization and slowed degradation of β-catenin may contribute to the robust signaling phenotype we observed. Future work from our lab will apply optogenetic tools to spatiotemporally manipulate pHi (58) while monitoring AJ protein dynamics.

Decades of research have been dedicated to understanding the dynamics and regulation of β-catenin in normal physiology and disease (8, 14). Our work provides new mechanistic insight in the context of pH-dependent differentiation, where stem cells have low pHi and high β-catenin abundance, and pHi increases are necessary for differentiation, accompanied by the loss of β-catenin (4, 59). Future work will explore roles for pHi dynamics in regulating β-catenin abundance through pH-dependent stability to support both maintenance of pluripotency (stabilized β-catenin at low pHi) and facilitation of differentiation (destabilized β-catenin at high pHi).

Finally, our work may shed light on how pHi regulates β-catenin abundance and transcriptional activity in cancer. Constitutively increased pHi is an emerging hallmark of cancer that is associated with several cancer cell behaviors (25). While pHi has been shown to increase with metastatic potential in tissue-matched cell lines (3), spatial pHi heterogeneity exists in 3D cancer cell cultures (60, 61). Concurrent work in our lab has revealed that microenvironment can alter pHi and concomitantly modulate β-catenin abundance and acquisition of vasculogenic mimicry phenotypes in cancer cell models (62). Thus, our work suggests that pHi changes may serve as a key physiological cue to regulate β-catenin abundance and activity to drive transcriptional and cell adhesion changes associated with cancer cell adaptation. More work is needed to simultaneously quantify pHi and β-catenin activity in more complex 3D cancer models to determine if pH-dependent regulation of the Wnt pathway (through β-catenin regulation) alters tumorigenic phenotypes. Our work characterizes pHi dynamics as a key regulator β-catenin abundance, AJ composition, and signaling function and provides a foundation to better understand context-specific avenues for pHi-dependent β-catenin abundance in regulating normal and disease biology.

## Experimental procedures

### Cell culture and conditions

MDCK cells were cultured in Eagle's minimal essential medium (Quality Biological, 112–018–101) supplemented with 10% fetal bovine serum (Peak Serum, cat: PS-FB3). Cells were maintained at 37 °C in a humidified incubator maintained at 5% CO_2_ (unless indicated otherwise). Cells were authenticated and tested for *mycoplasma* in November 2022 and again in December 2024.

### PHi manipulation

Cells were plated 24 h prior to being treated for pHi manipulation. Twenty-four hours after plating, the conditioned medium was aspirated and replaced with medium containing 5-(*N*-ethyl-*N*-isopropyl) amiloride) (Sigma, cat: A3085), an inhibitor of the sodium proton exchanger and S0859, a selective inhibitor of the sodium bicarbonate transporter (Sigma, cat: SML0638) 1 μM EIPA + 30 μM S0859 (Cf) (low pHi) or fresh medium and placed in an incubator maintained at 5% CO_2_ (low pHi and control) or 15% CO_2_ (high pHi) for 24 h. Where indicated, proteasome activity was inhibited 18 h after pHi manipulation treatment using 10 μM MG132 (Selleck, S2619) for a total of 6 h prior to sample analysis or collection.

### Acquisition and analysis of microscopy images

Plates were imaged using a Nikon Ti-2 spinning disk confocal microscope with a stage-top incubator (Tokai Hit), and a spinning disk confocal head (Yokogawa; CSU-X1), a Ti2-S-SE stage, multipoint perfect focus system, an Orca Flash 4.0 CMOS camera, four laser lines (405, 488, 561, 647 nm) with appropriate filter sets (DAPI: 405 nm laser excitation, 455/50 nm emission; GFP: 488 nm laser excitation, 525/36 nm emission; TxRed: 561 nm laser excitation, 605/52 nm emission; SNARF: 561 nm laser excitation, 705/72 nm emission; Cy5: 647 nm laser excitation, 705/72 nm emission) were used. Photoconversion experiments were performed with a digital micromirror device patterned illumination system (Polygon 400, Photometrix) and using a 405 LED. Immunofluorescent and live-cell transcriptional reporter images were collected on a 40X oil immersion objective (Nikon, CFI PLAN FLUOR NA 1.30). Photoconversion images were collected on a 60X oil immersion objective (Nikon, CFI PLAN APO OIL 1.40). Live-cell imaging was performed after 24 h of pHi manipulation. The stage top chamber was equilibrated to 37 °C and 5% CO_2_ (or 15% CO_2_ when capturing experimental images for high pHi) prior to loading plates on microscope.

### Trypan blue exclusion assay

MDCK cells were plated at 1 x 10^3^ cells per well in a 24-well plate. Cells were cultured for 24 h after plating before being treated for pHi manipulation as described above. After the 24-h treatment period, the medium was aspirated, the cells were washed with 1 ml prewarmed 1X Dulbecco’s phosphate-buffered saline (DPBS) (Quality Biological, cat: 114–057–101) prior to trypsinization. Cells were trypsinized in 100 μl of trypsin (Corning, cat: 25–053-CI) for 8 to 10 min to ensure detachment from the plates. Each well then received 100 μl prewarmed culture medium to inactivate the trypsin and resuspend the trypsinized cells to a single-cell suspension. Immediately before counting cell suspensions, 50 μl of the suspension was mixed with 50 μl trypan blue 0.4% solution (Gibco, cat: 15,250–061) in a separate Eppendorf tube. Cells were counted using 10 μl of the 1:1 cell suspension:trypan blue mixture in a hemocytometer for healthy (no dye uptake) and unhealthy/dying (blue) cells. Each biological replicate is the average of three wells (technical replicates) plated, treated, and counted on independent days.

### Population-level pHi measurement using 2′-7′-Bis-(2-carboxyethyl)-5-(and-)-carboxyfluorescein, acetoxymethyl ester

MDCK cells were plated at 1 x 10^3^ cells/ml in 24-well plate 48 h prior to performing the assay on the plate reader. Cells were treated for pHi manipulation as outlined above. After 24 h of treatment, 2′-7′-Bis-(2-carboxyethyl)-5-(and-)-carboxyfluorescein, acetoxymethyl ester (VWR/Biotium, 89139-244) was prepared as a 100 μM stock solution in 10% DMSO in DPBS. Cells were dye loaded with 1 μM 2′-7′-Bis-(2-carboxyethyl)-5-(and-)-carboxyfluorescein, acetoxymethyl ester for 30 min in respective culturing conditions. Prewarmed Hepes-based wash buffer (30 mM Hepes free acid, 145 mM NaCl, 5 mM KCl, 10 mM glucose, 1 mM MgSO_4_, 1 mM KHPO_4_, 2 mM CaCl_2_, pH 7.4) and Nigericin buffer [25 mM Hepes free acid, 105 mM KCl, 1 mM MgCl_2_ supplemented with 10 μM Nigericin (Fisher, N1495)] are prepared the day of the assay. Nigericin buffer is used to prepare three standard buffers with pH values ∼7.8, ∼7.2, and ∼6.5, where pH was accurately recorded to hundredths place for each point and each biological replicate for pHi back-calculation. After dye-loading, cells are washed 3 x 5 minutes with Hepes buffer containing the appropriate treatment at 37 °C. Experimental fluorescence intensities were measured on a Biotek Cytation5 plate reader (440ex/535em) and (490ex/535em) with kinetic reads taken every 39 s for 7 min (11 reads). Hepes wash buffer was aspirated and replaced with Nigericin buffer, pH ∼7.8 and incubated for 7 min at 37 °C before starting the kinetic read. This procedure was repeated for the other two Nigericin buffers. A data reduction step calculates the mean 490/440 intensity for experimental and standardized pH reads where slope and y-intercept are then calculated using Nigericin standard buffer pH values to the hundredth place. Population level pHi values are back-calculated using experimental ratios and Nigericin standard curve values.

### Whole-cell lysate preparation & protein quantification

After pHi manipulation (see above section), plates were removed from the incubator and placed on ice. The media was aspirated, and the plates were washed two times with ice-cold DPBS. Plates were wrapped with parafilm and stored at −80 °C if not prepared immediately. To wash or thaw plates on ice, 100 μl of lysis buffer [500 mM NaCl, 10 mM EDTA, 500 mM Hepes-free acid, 10 mM EGTA, 10% Triton X-100, and protease inhibitor cocktail (Pierce Protease Inhibitor Tablets, A32965; 1 tablet/50 ml lysis buffer), pH 7.4] was added to each plate. Cells were removed using cell scrapers and transferred to prechilled microfuge tubes, briefly vortexed, and then incubated on ice for 15 min. Lysates were centrifuged at 14,000 x g for 15 min at 4 °C. Clarified lysates were isolated to separate microfuge tubes on ice. The Bradford assay was used to quantify the total protein content of lysates (63) using Coomassie Protein reagent (Thermo Scientific, cat: 1856209). Lysates were stored at −80°C if not immediately being used for SDS-PAGE sample preparation.

### Immunoprecipitation

After pHi manipulation (see above sections), plates were removed from the incubator and placed on ice. The media were aspirated, and the plates were washed two times with ice-cold DPBS. For each plate, 250 μl of immunoprecipitation lysis buffer [50 mM Tris, 150 mM NaCl, 1 mM NaF, 1% Triton X-100, and protease inhibitor cocktail (Pierce Protease Inhibitor Tablets, A32965; 1 tablet/50 ml lysis buffer) (pH 7.5)] was added for 15 min on ice. Cells were scraped, and clarified lysate (10,000 rpm; 10 min) was used immediately. For E-cadherin immunoprecipitation, for each condition 100 μg of total protein was added to 2 μl of E-cadherin antibody (BD Bioscience, cat 610182) and incubated overnight at 4 °C with rotating. The next day, 40 μl of Protein G Dynabeads (Thermo Fisher, 10003D) was added to each sample and incubated for 2 h at 4 °C with rotating. Beads were separated with a magnet and washed (3 x 5 minutes) with PBS, separating with magnet between washes. For elution, 2X Laemmli (nonreducing, Bio-Rad 1,610,737) buffer was added to the bead volume, and beads were heated at 55 °C for 10 min before final magnetic separation. Eluted samples were then boiled for 10 min 100 °C with reducing agent (2-mercaptoethanol, 5% v/v) and then run on gels and transferred for immunoblotting (see Immunoblotting section for details).

### Immunoblotting

Protein samples were prepared in 6X Laemmli (reducing) buffer (Thermo Scientific, cat: J61337.AC) and boiled for 10 min at 100 °C. A total of 5 μg of MDCK lysate was added to each lane for immunoblot analysis. Protein samples were loaded onto 10% SDS-PAGE gels, which were run at 125V and 0.08 Amps for 15 min to stack samples. Protein samples were separated by running the gel at 175 V and 0.08 A for an hour and a half. Polyvinylidene difluoride (PVDF) membranes were presoaked in 100% ethanol for 5 min and rinsed 3X in water. Wet transfers were performed on ice at a constant amperage of 0.04 A (100V) for an hour and half in transfer buffer (Tris-Glycine buffer, 10% methanol, 0.01% SDS). Membranes were blocked for 1 h in 5% milk in 1X Tris-buffered saline + 0.1% Tween 20 (TBS-T) at room temperature with shaking. For phospho- β-catenin blots, membranes were blocked in 3% bovine serum albumin (BSA) in TBS-T. Membranes were washed 3 x 5 min in 1X TBS-T before being cut and incubated with primary antibodies. The following primary antibodies were used: β-catenin (BD Biosciences, 610,154; 1:5000 dilution in 3% BSA in 1X TBS-T), plakoglobin (Cell Signaling, 2309S; 1:2000 dilution in 3% BSA in 1X TBS-T), phospho β-catenin (pSer33/pSer37/pThr41) (Cell Signaling, 9561, 1:1000 dilution in 1% BSA in 1X TBS-T), non-phospho(active)-β-catenin (Ser33/Ser37/Thr41) (Cell Signaling, 8814, 1:1000 dilution in 1% BSA in 1X TBS-T), E-cadherin (Invitrogen, 13–1900, 1:1000 dilution in 3% BSA in 1X TBS-T), and actin (Santa Cruz, sc-58673; 1:500 diluted in 3% BSA in 1X TBS-T). Primary antibodies were incubated overnight at 4 °C with shaking. Primary antibody solutions were removed, and membranes were washed 3 x 5 minutes in 1X TBS-T before incubating secondary antibodies (Goat anti-mouse/rabbit HRP-conjugated antibodies; 1:10,000 diluted in 5% milk in 1X TBS-T) for 1 h at room temperature with shaking. Secondary antibody solutions were removed, and membranes were washed 3 x 5 minutes in 1X TBS-T before being developed using Pierce SuperSignal West Pico PLUS (Thermo Scientific, cat: 34580) (β-catenin, actin, and E-cadherin) or Pierce SuperSignal West Femto PLUS (Thermo Scientific, cat: 34095) (plakoglobin, phospho β-catenin, and all immunoprecipitation blots). Chemiluminescence and colorimetric images were acquired using a BioRad ChemiDoc imager.

### Western blot analysis

Raw files were exported from ChemiDoc imager and opened in ImageJ. Band intensities were acquired by densitometry analysis (area under the curve of individual bands in respective lanes). For each replicate blot, target protein intensities were normalized to the intensity of loading controls before normalizing each condition ratio to that of the control within biological replicates.

### Single-cell pHi calculations using SNARF-AM and confocal microscopy

Cells were plated at 5 x 10^3^ cells per well (4-well glass bottom dish, Matsunami, cat: D141400) or 2 x10^4^ cells per dish (35 mm dish with 14 mm glass cover slip, Matsunami, cat: D11130H) in complete medium 48 h prior to imaging. Cells were treated for pHi manipulation as described above. Fields of view (FOVs) were selected by viewing cells in differential interference contract (DIC). Cells were dye loaded in conditioned media with 10 μM of 5-(and-6)-Carboxy SNARF -1 Acetoxymethyl Ester, Acetate (Fisher Scientific, cat: C1272) prepared as a 100 μM stock solution dissolved in a 10% DMSO in DPBS for 10 min on the microscope stage. Dye-containing media were removed, and plates were washed three times with prewarmed complete growth medium containing appropriate treatment. A 10-min equilibration period was used prior to capturing images in SNARF (20% laser power, 400 ms), TxRed (20% laser power, 400 ms), and DIC (200 ms) channels for each FOV with five z-stacks ( ± 1 μm; 0.5 μm steps). SNARF dye was calibrated using three prewarmed Nigericin buffers (as described above). Complete medium was removed after the initial capture, and the pH ∼7.8 Nigericin buffer was used to wash the plate and incubated for 10 min prior to capture. This procedure was repeated for standard points at pH ∼7.2 and ∼6.5. After data acquisition, the NIS-Elements Analysis software was used to define single-cell ROIs after background subtraction for each channel. The mean fluorescence intensity for each ROI in each channel was exported to Microsoft Excel. Ratios were calculated by dividing the SNARF mean intensity by the TxRed mean intensity for each capture. The three ratios for each nigericin point and their corresponding pH values were used to calculate slope and y-intercept. These values and the experimental ratio of SNARF/TxRed were used to back calculate single-cell pHi values.

### Immunofluorescent staining

Cells were cultured in 35 mm dish with 14 mm glass cover slip (Matsunami, cat: D11130H) and plated for experiments 48 h prior to fixation. 24 hours after plating, pHi was manipulated as described above. After 24 hours of pHi manipulation, cells were washed with ice-cold DPBS then fixed in 3.7% paraformaldehyde in DPBS for 10 min at room temperature with shaking. The fixative solution was removed, and cells were washed 3 x 2 minutes in DPBS. Cells were permeabilized for 10 min at room temperature in a 0.1% Triton X-100 in 1X DBPS. Permeabilization buffer was removed and washed 3 x 2 minutes before blocking for 1 h at room temperature in a 1% BSA (Millipore Sigma, cat: 2910-OP) in DPBS solution. The blocking solution was removed and washed 3 x 2 minutes in DPBS. Primary antibodies (see Immunoblotting methods 1.3.7 for vendor information) were diluted in antibody solution (1% BSA, 0.1% Triton X-100 in DPBS) with the following dilutions: mouse anti-β-catenin (1:200), rabbit anti-plakoglobin (1:400), rabbit anti-non-phospho-β-catenin (1:100), and rat anti-E-cadherin (1:200). Primary antibody solutions were incubated in dishes at 4 °C overnight with rocking. After primary antibody incubation, the plates were washed 3 x 2 minutes prior to incubating Alexa Fluor-conjugated secondary antibodies diluted 1:1000 in antibody solution for 1 h at room temperature while being protected from light. Secondary antibody solutions were removed and washed from the plates 3 x 2 minutes using DPBS. Cells were counterstained with Hoechst dye (DAPI; Thermo Scientific, cat: 62249) diluted 1:10,000 in antibody solution for 10 min at room temperature protected from light. Counterstain was removed and washed 3 x 2 minutes in DPBS. Imaging dishes were stored and imaged in DPBS. Image acquisition details are outlined above. Z-stacks were collected 2 μm above and below a relative z-plane that captured the center of the cell determined using the DAPI channel. Nuclear pools of proteins were identified using Nikon Elements Analysis software by autodetecting individual ROIs in the DAPI channel that represent individual nuclei. Whole cell ROIs were hand drawn using E-cadherin as a membrane marker to differentiate single cells, drawing the ROI interior to E-cadherin staining to prevent inclusion of membrane signal. Nuclear ROIs were subtracted from hand-drawn whole cell ROIs to produce cytoplasmic ROIs, excluding both cell membranes and nuclei. The mean intensity for each channel was exported for each z-plane that had the largest mean intensity in the DAPI which indicated the center of the individual cells. Junctional protein levels were quantified using IMARIS software (Oxford Instruments, version 9.5.1) by generating surfaces based on E-cadherin intensity. Mean intensities for all channels within each surface were exported and analyzed for statistical significance using GraphPad Prism software.

### Quantifying cell area and cell volume

MDCK cells treated with pHi manipulation conditions were labeled with CellMask Deep Red (Thermo Fisher, C10046; 1:20,000) for 15 min. Cells were then imaged with Z-stacks collected encompassing the entire cell volume. For cell area calculation, a single z-plane from the center of the cell was analyzed in IMARIS. Individual cells were identified using the cells module based on the CellMask signal (cell membranes), and cell areas were exported. For cell volume calculations, a surface was generated in IMARIS using the CellMask signal, and the complete Z-stack, filtration settings were adjusted such that the smallest cell diameter detected was 12.0 μm, and cell membrane detail was set to 1.2 μm before cell volumes were exported. Area and volume data were analyzed for statistical significance using GraphPad Prism software.

### Cycloheximide chase assay

Cells were plated at 1.5 x 10^4^ cells per well in 6 well plate 48 h prior to collecting lysates. Twenty-four hours after plating, cells were treated for pHi manipulation as described above. Stock solutions of cycloheximide (VWR, 9427) were prepared fresh for each experiment. After 20 h of pHi manipulation, all plates except time-0 samples were treated with 50 μg/ml cycloheximide and returned to the appropriate incubator. Immediately after treatment, lysates from time-0 plates were prepared as described previously, and the remaining samples were lysed every 30 min for 2.5 h. Immunoblotting and analysis of β-catenin and actin were performed as described above. The ratio of β-catenin to actin for each time point was normalized to time = 0 h within each condition and for each replicate. Normalized data for each biological replicate was imported to Prism where one-phase decay was used to determine half-life values.

### Quantifying protein degradation and relocalization using mMaple3 constructs via photoconversion and confocal microscopy

MDCK cells were seeded at 3 x 10^6^ cells in 10 cm dishes and allowed to adhere to the dish for 8 h after seeding, and cells were transfected with 15 μg mMaple3 containing plasmid (mMaple3 alone, mMaple3-β-catenin WT, or mMaple3- β-catenin-H36R) using Lipofectamine 3000 according to the manufacturer’s instructions. Transfections proceeded for 18 h prior to the removal of transfection media, rinsing of the plate with 10 ml of warm DPBS, and addition of 1 ml trypsin. Cells were trypsinized at 37 °C for 8 to 10 min. After all the cells were detached, complete medium was added to the dish to create a single cell suspension. Using the transfected single cell suspension, 35 mm imaging dishes were seeded at 2.5 x 10^4^ cell per dish and adhered for 8 h. After the cells adhered for 8 h, pHi manipulation was performed as described above. Images were collected as described above. Cell nuclei were visualized *via* SPY650-DNA (Cytoskeleton, cat. #: CY-SC501) probe that was incubated for 2 h at 37 °C at a 1:1000 dilution in complete media. Photoconversion was performed using a digital micromirror device patterned illumination system (Polygon 4000) with 405 nm LED set to 100% power. For each experiment, an extra plate was prepared to calibrate the digital micromirror device for a fixed stimulation ROI with a conserved area across cells and conditions (*i*.*e*. 6 x 16 pixels). Prestimulation images were acquired using GFP (preconversion mMaple3; 30% laser power, 500 ms) and Cy5 (DNA; 25% laser power, 200 ms), which were used to place the stimulation ROI. Within the Nikon Elements software, an ND stimulation protocol was used to stimulate at 405 nm within a user-defined stimulation ROI with a conserved area across cells and conditions (*i*.*e*.*,* 6 x 16 pixels) and then acquire images across the entire FOV using TxRed (converted mMaple3; 30% laser, 500 ms). For these experiments, a preconversion image was acquired followed by photoconversion in the stimulation ROI of mMaple3 or mMaple3-β-catenin constructs using 405 nm light at 100% LED power for 90 s. Then, the converted (TxRed) signal was monitored every 30 s for 10 min postconversion. ROIs for nuclei and cytoplasm were generated using GFP (unconverted mMaple3) and Cy5 (DNA probe) images and applied to the photoconverted movies to track nuclear and cytoplasmic signal over time.

Cytoplasmic tracking data were exported to excel and normalized to the first capture following photoconversion. Single-cell traces were then fit using nonlinear regression, comparing one-phase and two-phase decay curve fits with a constrained Y_o_ equal to 1.0. For all conditions and proteins, two-phase decay fit the data more accurately but revealed similar values for the fast rate of decay for mMaple3 alone, mMaple3-β-catenin WT, and mMaple3- H36R-β-catenin, indicating this represents diffusion and/or bleaching. Single cell curves were then normalized to the time point after photoconversion that represented the start of the slow decay curves. This normalization accounts for the initial loss of fluorescent signal due to photobleaching and diffusion that was consistent across construct and conditions to enable curve fitting of protein stability. All reported half-life values are from the slow phase decay fits. The same two-phase decay models were used to compare mMaple3-β-catenin degradation under low, control, and high pHi conditions. Nuclear relocalization was quantified by generating nuclear ROIs based on SPY650-DNA intensity (see above) and quantifying nuclear intensity over the same time course used to monitor cytoplasmic degradation, in matched cells. Nuclear intensity was normalized to t = 185 s, and median traces were imported to Prism and fit using nonlinear regression and a one-phase association model with a constrained Y_o_ equal to 1.0 to account for the normalization we performed. Overall nuclear accumulation was readout by comparing plateau values between pHi conditions, where statistical comparison demonstrated that the same plateau value cannot adequately fit each dataset.

### Single-cell β-catenin transcriptional activity assays using live-cell microscopy

Lentiviral-TOP/FOP-dGFP-reporters (wildtype consensus plasmid TOP: Addgene plasmid #14715; http://n2t.net/addgene:14715; RRID:Addgene_14715; inverted consensus plasmid FOP: Addgene plasmid # 14885; http://n2t.net/addgene:14885; RRID:Addgene_14885) were a gift from Tannishtha Reya. For single-cell transcriptional assay validation, we conducted preliminary experiments using transient transfections. Cells were seeded at 3 x 10^6^ cells in 10 cm dishes and allowed to adhere to the dish for 8 h after seeding, and cells were transfected with 0 μg (mock transfection) or 15 μg TOP-dGFP-reporter/FOP-dGFP-reporter plasmid using Lipofectamine 3000 according to the manufacturer’s instructions. Transfections proceeded for 18 h prior to the removal of transfection media, rinsing of the plate with 10 ml of warm DPBS, and addition of 1 ml trypsin. Cells were trypsinized at 37 °C for 8 to 10 min. After all the cells were detached, complete medium was added to the dish to create a single cell suspension. Using the transfected single cell suspension, 35 mm imaging dishes were seeded at 1.5 x 10^4^ cell per dish and adhered for 8 h. After the cells adhered for 8 h, the plating medium was removed and replaced with complete media used for pHi manipulations (see section above) or Wnt stimulation [40 ng/ml (Cf) murine Wnt3a (Peprotech; cat: 315–20)].

After validating the reporter, a stable cell line was generated. First, the TOP-dGFP reporter plasmid was transfected into MDCK cells. Briefly, single-cell dilutions of transfected MDCK cells were plated at low density (50 cells/ml) in 96-well plates. Colonies were tested for TOP-dGFP expression in a Wnt stimulation protocol [with and without 1000 ng/ml murine Wnt3a (Peprotech; 315–20)]. A final clone was chosen that had robust reporter response and with matched morphology and pHi of parentals. Cells were seeded at 1.5 x 10^4^ cell per 35 mm imaging dish and adhered for 8 h. After adherence, plating medium was removed and replaced with complete media for pHi manipulations (see section above) with or without Wnt stimulation [0 ng/ml to 1000 ng/ml (Cf) murine Wnt3a (Peprotech; 315–20)].

For all single-cell Wnt stimulation assays, images were acquired as outlined in the above sections. Cell nuclei and cell membranes were visualized *via* Hoechst dye (DAPI; Thermo Scientific, cat: 62249; 1:10,000) and CellMask Deep Red (Thermo Fisher, C10046; 1:20,000), respectively, incubated for 15 min at 37 °C in complete media. Fields of view were selected by visualizing nuclei (DAPI), and images were collected in the DAPI (20% laser power, 400 ms), GFP (30% laser power, 500 ms), Cy5 (20% laser power, 400 ms), and DIC (32.6% DIA, 200 ms) channels. Whole-cell ROIs were drawn within individual cells using cell mask as a membrane marker, and the average GFP intensity for individual cells were exported to Excel. After stable cell line of TOP-dGFP, all cells were analyzed for GFP fluorescence intensity. For transient transfections, single-cell intensities that were greater than 2X, the average intensity of mock-transfected cells were imported to GraphPad Prism for statistical analysis and visualization. None of the cells expressing FOP-dGFP from the transient transfections met the 2-fold mock cutoff, indicating the robust specificity of this reporter assay.

### Structural preparation, pKa analysis, and metrics determination

Existing high-resolution structures of β-catenin bound to β-TrCP were obtained from the PDB: 1P22 (44), 6M94 (45), as well as a high-resolution ternary structure of 6M94 (45), . We also generated AlphaFold (46) structures of both doubly phosphorylated β-catenin (pSer33/pSer37) and doubly phosphorylated plakoglobin (pSer24/pSer28). We input just the WD40 repeats of β-TrCP (residues 275-end) and just the N-terminal region of β-catenin or plakoglobin (residues 1–100 for both). Resulting AlphaFold structures and available crystal structures were submitted to PROPKA3 (64), a computational program that predicts residue pKas and electrostatic interactions based on 3D structural data. From these output data, we reported features of the structures that we predict are most relevant for β-TrCP binding (pKa, %Buried, coulombic interactions). We used Pymol to calculate heteroatom distances where indicated.

### Evolutionary conservation analyses

We performed NIH protein–protein Blast search on residues 1 to 100 of human β-catenin and filtered for the top 1000 hits. While not exhaustive, the goal was to indicate the comparative proportions of histidine- and nonhistidine-containing phosphodegrons. Consensus motifs ranging from −5 to +5 residues around the DSGXHS motif were obtained from the top 1000 BLAST hits to human β-catenin. These sequences were uploaded into the WebLoGo web software (https://weblogo.berkeley.edu/logo.cgi) (65). We also generated sequence logos for this dataset output filtered separately by protein hit identity (β-catenin or Plakoglobin). The resulting amino acid sequence consensus motifs are included in Fig. S10. Table S1 was generated based on hits of the BLAST search with an additional filtration: only BLAST hits with an intact DGSXXS motif were included in the analysis. Sequences were then grouped by phosphodegron motif as indicated in the table headers.

### Statistics

GraphPad Prism (10.0) was used to prepare graphs and perform statistical comparisons as indicated in each figure legend. Normality tests were performed on all datasets after performing outlier analysis on pooled data (ROUT method; Q = 1%), where normally distributed data are shown with means, and non-normally distributed data are shown with medians. “N” indicates the number of biological replicates performed, and “n” represents the number of technical replicates or individual cell measurements collected.

## Data Availability

The data associated with this work are included in this article and the supporting information file. Raw data are available upon request from the corresponding author.

## Supporting information

This article contains supporting information.

## Conflict of interest

The authors declare that they have no conflicts of interest with the contents of this article.
