# Supplementary material for: Intracellular pH regulates β-catenin with low pHi increasing adhesion and signaling functions
Source: J Biol Chem. 2026 Jan 7;302(3):111134. doi: 10.1016/j.jbc.2026.111134 (PMC12907494; doi:10.1016/j.jbc.2026.111134)
Supplement: Supplementary Material 1 [file mmc1.pdf]

Supplementary Video 1. **Representative cytoplasmic photoconversion experiment of MDCK cells expressing mMaple3-β-catenin.** Scale bar = 20 μm. Video shown in inverse mono with stimulation ROI (red rectangle) overlaid each frame. The first frame represents pre-photoconversion image in channel to visualize converted mMaple3. The second frame shows converted mMaple3-β-catenin after 90 seconds of stimulation with 405 nm light within stimulation ROI with subsequent images collected over a 10-minute tracking period.

Supplementary Table S1. Conservation of β-TrCP phosphodegron motifs. We performed NIH protein-protein Blast search on residues 1-100 of human β-catenin and filtered for the top 1,000 hits. Table S1 was generated based on hits of the BLAST search with an additional filtration: only BLAST hits with an intact **DGS**XX**S** motif were included in the analysis. Headers show motif identity, rows are accession numbers.

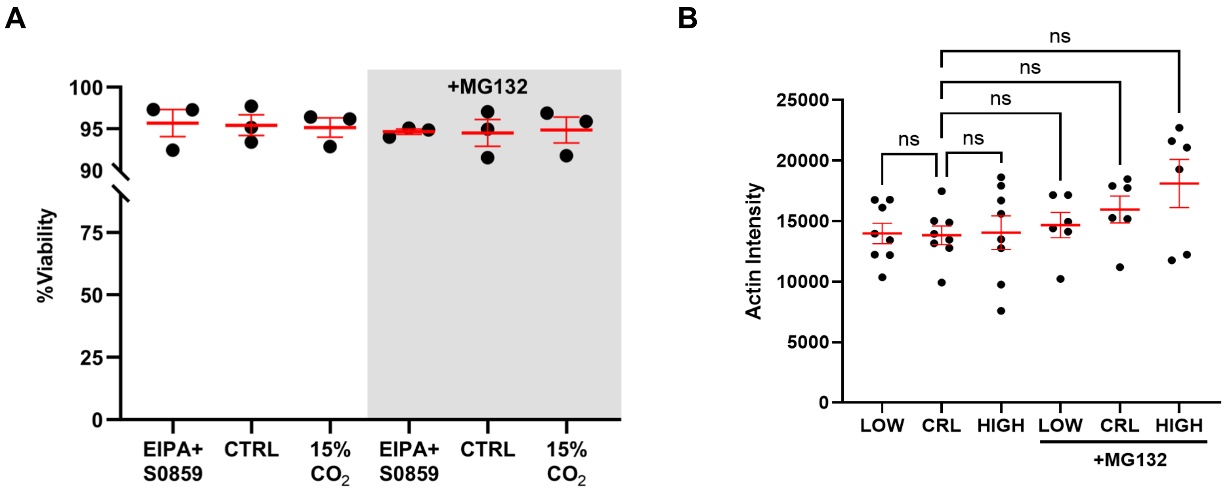

Figure S1. Methods for manipulating intracellular pHi do not affect cellular viability or protein abundance of loading control proteins (Actin). (A) Cellular viability as determined by trypan blue exclusion assay (see Methods). Means from 3 biological replicates were calculated from technical triplicates. Mean and SEM are shown. **(B)** Quantification of actin intensity from immunoblots prepared as in Figure 1. Mean and SEM are shown from 6-9 biological replicates. For both A and B, statistical significance was determined using one-way ANOVA test with Sidak's correction for multiple comparisons.

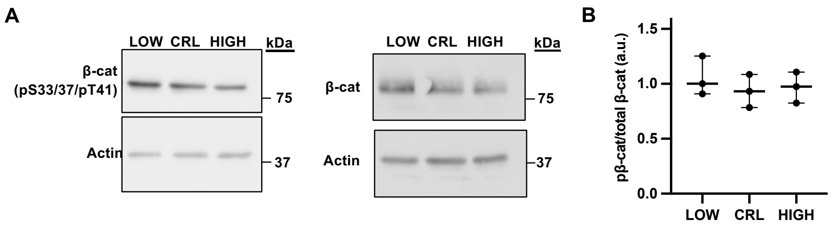

Figure S2. Levels of phosphorylated β-catenin are not significantly altered by pHi in whole cell lysates. (A) Representative immunoblot of β-catenin (β-cat), phospho-β-catenin (pSer31/pSer33/pThr41) and actin under low, control, and high pHi conditions in MDCK cells. **(B)** Quantification of β-catenin immunoblot data collected as described in A. Individual biological replicates were normalized to control MDCK within each experiment. Scatter plots show all data points from n=4 biological replicates. Statistical significance was determined using a ratio paired t-test between treatment groups and one-sample t-tests with a hypothetical mean of 1.0 when comparing to control (which had no variation due to normalization).

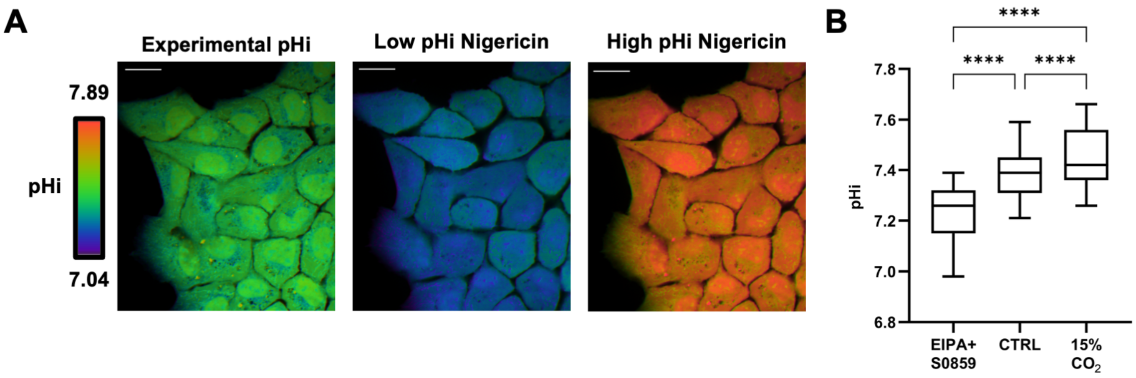

Figure S3. 24-hour pHi manipulation conditions significantly alter pHi at the single-cell level. (A) Representative confocal microscopy images of MDCK cells for pHi measurements and nigericin standardization (see Methods). Ratiometric display of emission at 640nm/580nm with 488 nm excitation. Scale bars: 20 µm. **(B)** Box and whisker plots of back calculated pHi (EIPA+S0859, n=244; CTRL, n=299; 15% CO_2, n=204; from two biological replicates). Medians are shown using Tukey display. Significance was determined by the Kruskal-Wallis test with Dunn's multiple comparisons correction. ****$P<0.0001$.

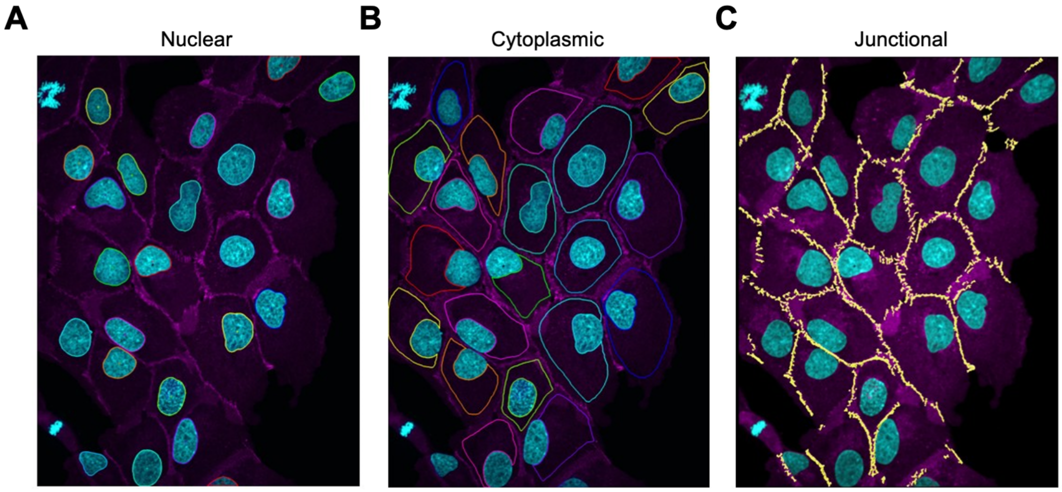

Figure S4. Analysis pipeline for quantifying protein abundance in various subcellular pools. (A) Nuclear regions of interest (ROIs) were generated by autodetecting single-cell nuclei based on Hoechst staining. **(B)** Cytoplasmic ROIs were created by subtracting nuclear ROIs (A) from whole cell ROIs by drawing interior to cell boundaries marked by E-cadherin staining (magenta). **(C)** 3D junctional surfaces were generated from z-stacks and thresholded based on E-cadherin (magenta) staining.

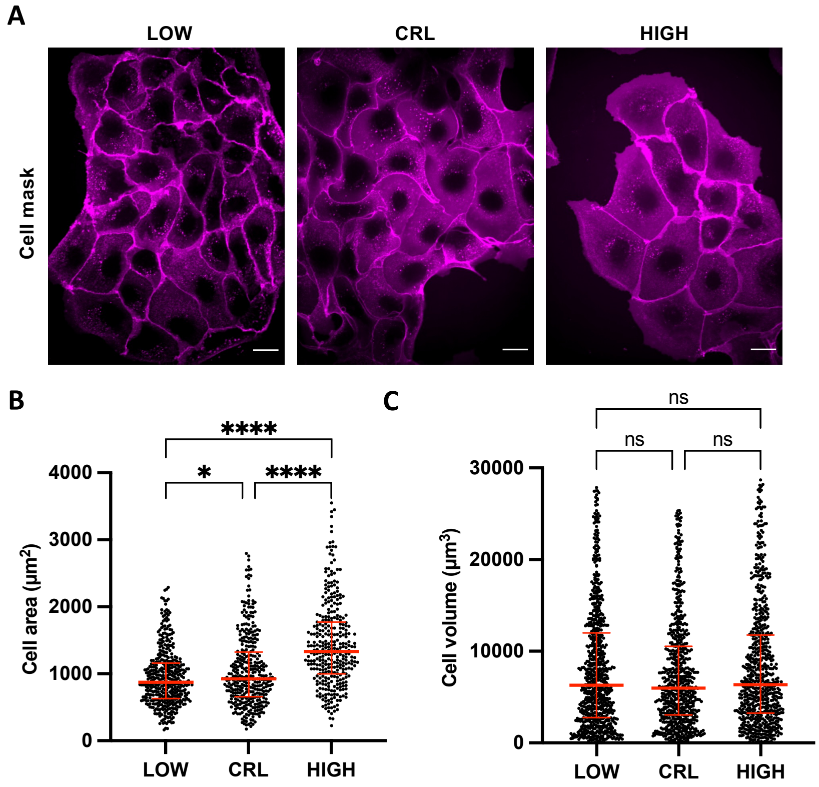

Figure S5. Cell morphology analysis shows pH-dependent cell area changes but unchanged cell volume. (A) Representative images of MDCK cells with low, control, and high pHi with membranes stained with CellMask Deep Red (see methods). Scale bars, 20 μm. **(B)** Quantification of single cell area for MDCK cells prepared as in A. Scatter plots of individual cells, median and interquartile range shown in red. N=3 biological replicates. **(C)** Quantification of single cell volume for MDCK cells prepared as in A. Scatter plots show individual cells, median and interquartile range shown in red. N=3 biological replicates. For B and C, significance was determined by the Kruskal-Wallis test with Dunn's multiple comparisons correction. *P<0.05, ***P<0.0001.

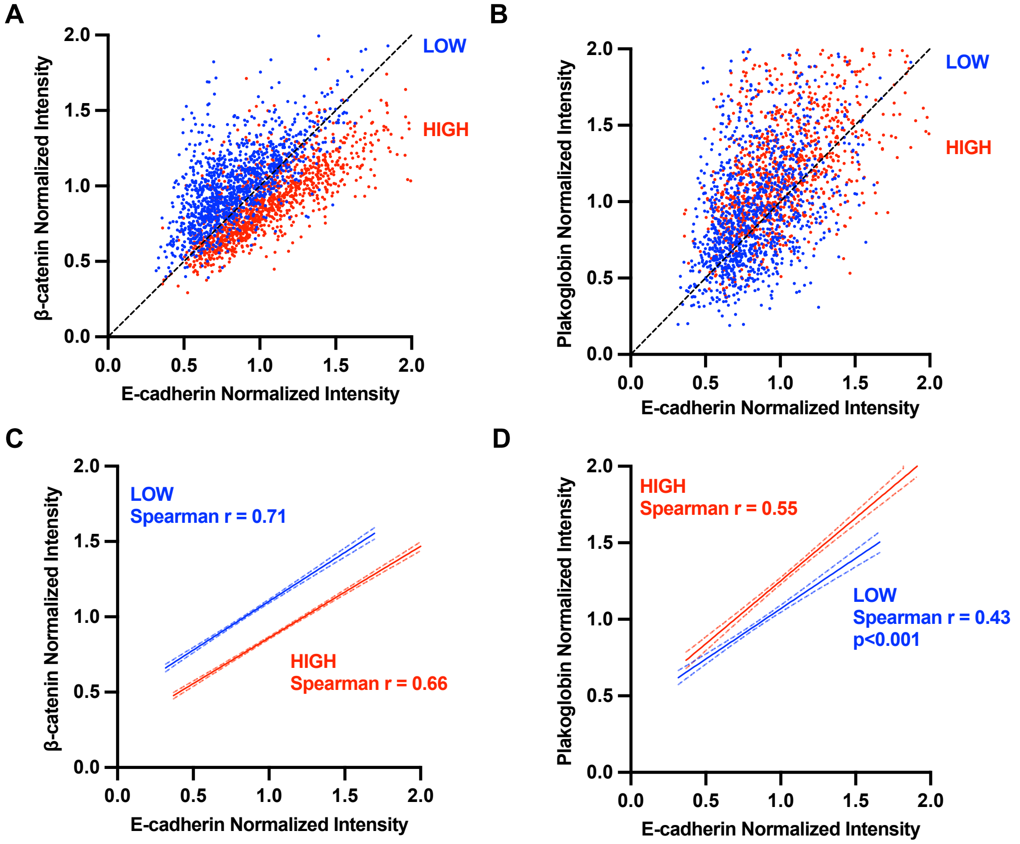

Figure S6. Correlation of junctional E-cadherin, β-catenin, and plakoglobin. Correlation plots from immunofluorescent data obtained from MDCK cells at low and high pHi co-stained with E-cadherin, β-catenin and plakoglobin. **(A-B)** Data shown are normalized fluorescence intensities of **(A)** β-catenin vs. E-cadherin and **(B)** plakoglobin vs. E-cadherin. From 3 biological replicates. All data were normalized to median values from control MDCK within each biological replicate. Dotted lines indicate identity (x=y). **(C-D)** Simple linear regressions shown with 95% confidence intervals. C is from data in A and D is from data in B. Shown in insets are Spearman correlation coefficients (r) for the data. P values indicated for statistically significantly different correlations when comparing across pHi.

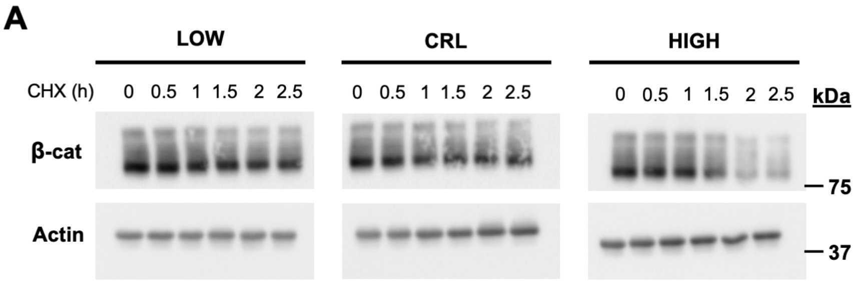

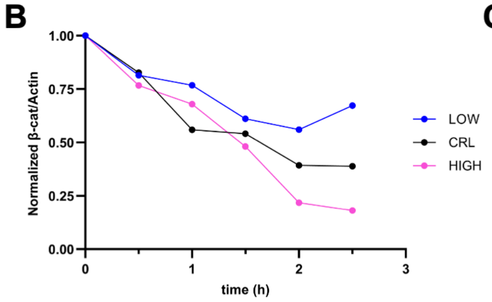

C

Condition	Half-life (h)	R^2
LOW	2.79+0.03	0.75
CRL	1.58+0.03	0.96
HIGH	1.22+0.03	0.94

Figure S7. Intracellular pH regulates the half-life of endogenous β-catenin. (A) Representative immunoblots of β-catenin and actin. Cells were treated for pHi manipulation for 24 hours before being treated with cycloheximide (CHX) for the indicated amounts of time. **(B)** Quantification of immunoblots in (A) where β-catenin levels were normalized to actin. Ratios were normalized to t=0 h, plotted and fit with nonlinear regression. From one biological replicate. **(C)** Half-life values obtained using one-phase decay curve fitting for data in (B).

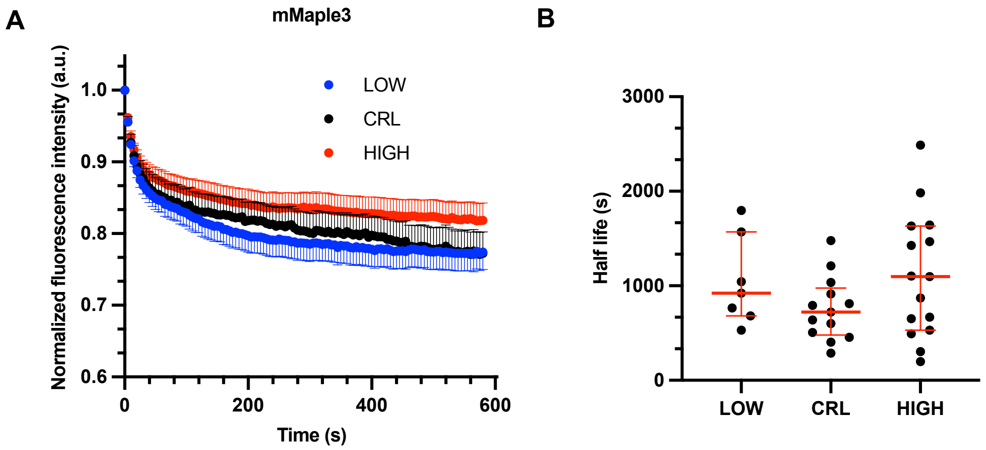

Figure S8. mMaple3 has pH independent degradation. (A) Normalized fluorescence intensity traces from MDCK cells expressing mMaple3 under low, control, and high pHi conditions. Dots show mean cell traces, error bars indicate SEM. Reported half-life calculated from mean cell traces. LOW, n=11; CRL, n=14; HIGH, n=18 from 3 biological replicates. **(B)** Single-cell half-life values were obtained by fitting individual single-cell traces from cells collected as in A. (median±IQR) Outliers were removed using the ROUT method (Q=1%). LOW, n=6; CRL, n=13; HIGH, n=15 from 3 biological replicates. Statistical significance in C was determined using Kruskal-Wallis test with Dunn’s correction for multiple comparisons, no statistically significant differences were identified.

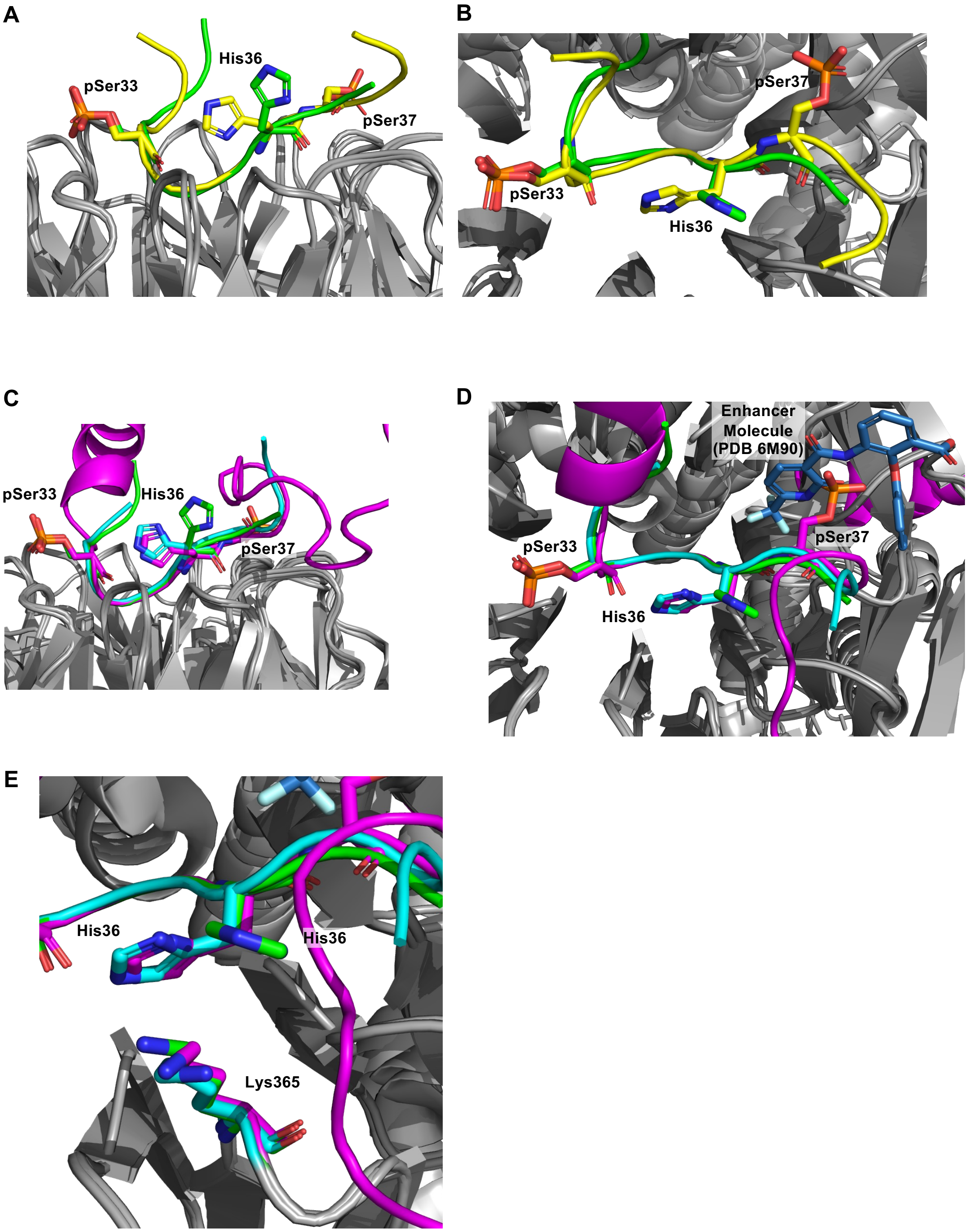

F

Structural Information						Calculated properties			
Structure	Obligate Phosphorylation		Other ligand	pH (of crystallization or prediction)	Predicted β-TrCP binding affinity	His36 pKa	His36 % Buried	His36-Lys365 Coulombic Interaction	His36-pSer33 Coulombic Interaction
	pSer33	pSer37							
6M94	Yes	No	No	Initial crystalization 5.5, soaked at pH 7.1	Low	5.79	23%	-0.36	N/A
6M90	Yes	No	Yes	Initial crystallization 5.5, soaked at pH 7.1	Medium	5.26	59%	-0.39	0.46
1P22	Yes	Yes	No	6.8	Medium	4.6	58%	-0.45	0.56
Alphafold	Yes	Yes	No	7.2	High	4.7	85%	-0.49	0.53

Figure S9. Analysis of β-catenin and β-TrCP crystal structures. (A) Structures of β-TrCP (grey) with medium affinity β-catenin (pSer33/pSer37, low pH) (PDB: 1P22, yellow) and low affinity β-catenin (pSer33/Ser37, low pH) (PDB: 6M94, green)**.** His36, pSer33 and pSer37 (if present) are shown in stick. **(B)** Top-down view of structures shown in A. **(C)** Structures of β-TrCP (grey) with low affinity β-catenin (pSer33/Ser37, low pH) (PDB: 6M94, green), medium affinity β-catenin (pSer33/Ser37, small molecule binder, low pH) (PDB: 6M90, cyan), and high affinity β-catenin (pSer33/pSer37, high pH) obtained from Alphafold (magenta)**.** His36, pSer33 and pSer37 (if present) are shown in stick. **(D)** Top-down view of structures shown in C. In this view, the small molecule binder is included in the 6M90 structure display and shown in blue stick. **(E)** Interactions of His36 of β-catenin and Lys365 of β-TrCP. His36 and Lys365 are shown in stick, with coloring matching parent structure (PDB: 6M94, green), (PDB: 6M90, cyan), and Alphafold structure (magenta). **(F)** Table of structure details and calculated structural properties.

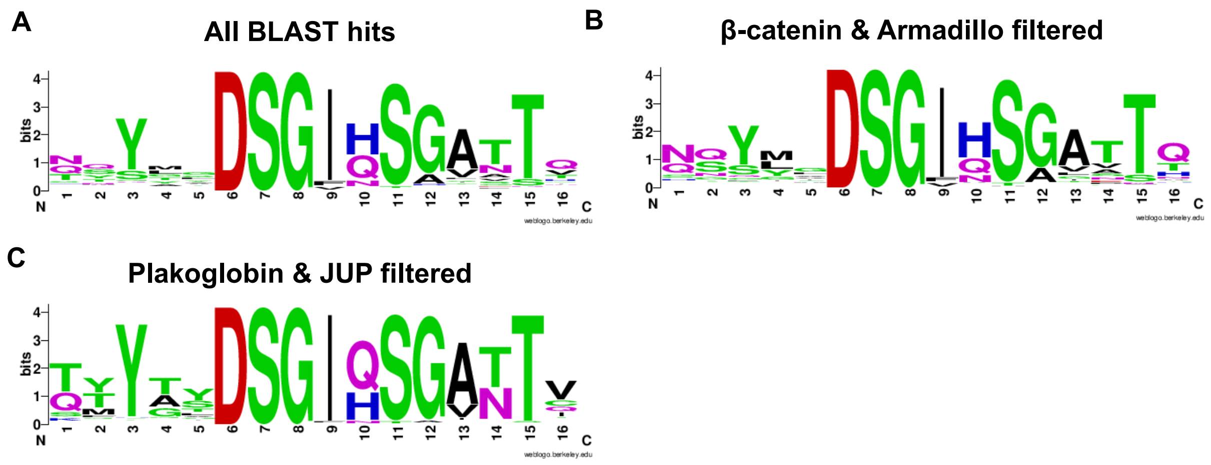

Figure S10. Comparison of β-TrCP substrate phosphodegron consensus motifs. (A) WebLoGo of phosphodegron motif for top 1,000 blast hits against human β-catenin **(B)** Hits from A further filtered for “β-catenin” or “armadillo” **(C)** Hits from A further filtered for “plakoglobin” or “JUP”.
